# Effects of rhizosphere fungi on the chemical composition of fruits of the medicinal plant *Cinnamomum migao* endemic to southwestern China

**DOI:** 10.1186/s12866-021-02216-z

**Published:** 2021-07-06

**Authors:** Jingzhong Chen, Xiaolong Huang, Bingli Tong, Deng Wang, Jiming Liu, Xiaofeng Liao, Qingwen Sun

**Affiliations:** 1 Forest Ecology Research Center, College of Forestry, Guizhou University, Guiyang, 550025 Guihzou Province China; 2 Guizhou province Institute of Mountain Resources, Guiyang, 550025 China; 3 College of Pharmacy, Guizhou University of Traditional Chinese Medicine, Guiyang, 550025 China

**Keywords:** *Cinnamomum migao*, Small watershed, Fruit period, Rhizosphere fungi, Chemical components

## Abstract

**Background:**

This study examined how rhizosphere fungi influence the accumulation of chemical components in fruits of a small population species of *Cinnamomum migao*.

**Results:**

Ascomycota and Basidiomycota were dominant in the rhizosphere fungal community of *C. migao*. *Pestalotiopsis* and *Gibellulopsis* were associated with α-Terpineol and sabinene content, and *Gibellulopsis* was associated with crude fat and carbohydrate content. There were significant differences in rhizosphere fungal populations between watersheds, and there was no obvious change between fruiting periods. *Gibberella, Ilyonectria*, *Micropsalliota*, and *Geminibasidium* promoted sabinene accumulation, and *Clitocybula* promoted α-Terpineol accumulation.

**Conclusion:**

The climate-related differentiation of rhizosphere fungal communities in watershed areas is the main driver of the chemical composition of *C. migao* fruit. The control of the production of biologically active compounds by the rhizosphere fungal community provides new opportunities to increase the industrial and medicinal value of the fruit of *C. migao*.

**Supplementary Information:**

The online version contains supplementary material available at 10.1186/s12866-021-02216-z.

## Background

The rhizosphere represents the interface of plant-soil interactions and was first proposed by Hiltner in 1904. The rhizosphere includes the microenvironment 0 ~ 2 mm between the root surface and soil [24] and represents the node of energy exchange between plant and soil. Interactions in the rhizosphere alter the physical, chemical, and biological characteristics of the soil [31]. The diversity and function of rhizosphere fungi are often related to root exudates (proteins and sugars), and many organic compounds secreted by plant roots provide energy for and induce greater densities of rhizosphere fungi [50]. Under natural conditions, rhizosphere fungi form a beneficial symbiotic relationship with most plants, the rhizosphere fungal providing nitrogen and phosphorus in return for their hosts, and can significantly promote the utilization efficiency of soil nutrients by plants [53]. The influence of rhizosphere fungi on plant growth and development has been widely studied, particularly in the context of improving plant productivity and crop yields [51]. Many studies have shown the influence of rhizosphere fungi on the chemical composition of medicinal plants [11, 77]. Indeed, fungal communities in the rhizosphere promote the accumulation of beneficial substances in medicinal parts, with some fungi directly secreting plant growth hormones or other important metabolites [4, 27, 32].

*C. migao* is a large evergreen of the Lauraceae family and is endemic in southwest China. It is only distributed in the dry and hot valleys formed by the Yujiang, Panjiang, and Hongshui Rivers at the border of the provinces of southwestern Yunnan, Guizhou, and Guangxi [39]. *C. migao* fruit is rich in metabolites such as sugar, crude fat, and volatile oils [69, 78]. Among the Miao and Zhuang populations in southwestern China, *C. migao* fruit is used as a seasoning and as traditional herbal medicine [76]. Pharmacological studies have shown that *C. migao* fruit effectively treats gastrointestinal tract and cardiovascular diseases [38, 70, 71, 74]. Indeed, four patent medicines in China contain *C. migao* fruit as the main ingredient and have achieved sales of hundreds of millions of RMB. *C. migao* fruit is only distributed in the narrow region on the borders of the Panjiang, Yujiang, and Hongshui Rivers with an area of about 160,000 km^2^ in southwestern China and is cataloged in the *List of Red Species of Biodiversity in China-Volume of Higher Plants* as near-endangered [47]. Studies have confirmed the considerable geographical variation in the chemical composition of *C. migao* fruit despite the narrow regional distribution, and the variety is not genetically derived. Researchers have suggested that the chemical components of *C. migao* fruit may be closely related to the climate of the small watershed in the plant’s distribution region [9, 79]. Karst landforms characterize the geography of southwest China; this special geomorphic discontinuity has formed many small watersheds with different climates [19]. The climate differences formed by this special geographic area also promoted species differentiation and rich species diversity, including the ecological differentiation of fungal populations to form special ecologically functional populations [42, 62]. Extensive studies confirmed that environmental differences affect the feedback mechanism between plants and rhizosphere fungi, which then influence plant growth and chemical characteristics [37, 54, 70, 71]. We aimed to understand how climatic differences between small watersheds affect the composition and function of the rhizosphere fungal community of *C. migao* and the chemical composition of the fruit. This study was conducted with the aim of answering the following research questions: How do environmental differences in small watersheds and different fruiting periods affect the composition and function of the fungal community in the rhizosphere of *C. migao*? How do differences in the community composition and function of *C. migao* rhizosphere fungi affect the feedback mechanism between *C. migao* and rhizosphere fungi and further affect the accumulation of fruit chemical components?

In order to explore the influence of special rhizosphere fungal communities on the chemical composition of *C. migao* in a small watershed climate, we studied the distribution of *C. migao* in small watersheds (Yujiang, Panjiang, and Hongshui Rivers) and different fruiting periods (young fruit period, closed immature period, maturity period). We also explored changes in the rhizosphere fungal community and the relationship between the climate of the small watershed and the chemical properties of the rhizosphere soil. We used high-throughput sequencing to characterize the fungal communities and measured the dynamic changes in the chemical composition of *C. migao* fruit. The results showed that the rhizosphere fungi affected the *C. migao* fruit content of carbohydrate, crude fat, α-terpineol, and sabinene. The fungal communities significantly differed between watershed areas but not between fruiting periods. We observed correlations and dynamic models that explain to a certain extent that the microclimate-dependent rhizosphere fungal community may be the cause of the geographic variation in the chemical composition of the fruit. These findings provide a feasible strategy for improving the medicinal value of the fruit of *C. migao* by controlling the rhizosphere microbiome.

## Results

### Alpha diversity and fungal composition of rhizosphere soil fungi

**Fig. 1:**
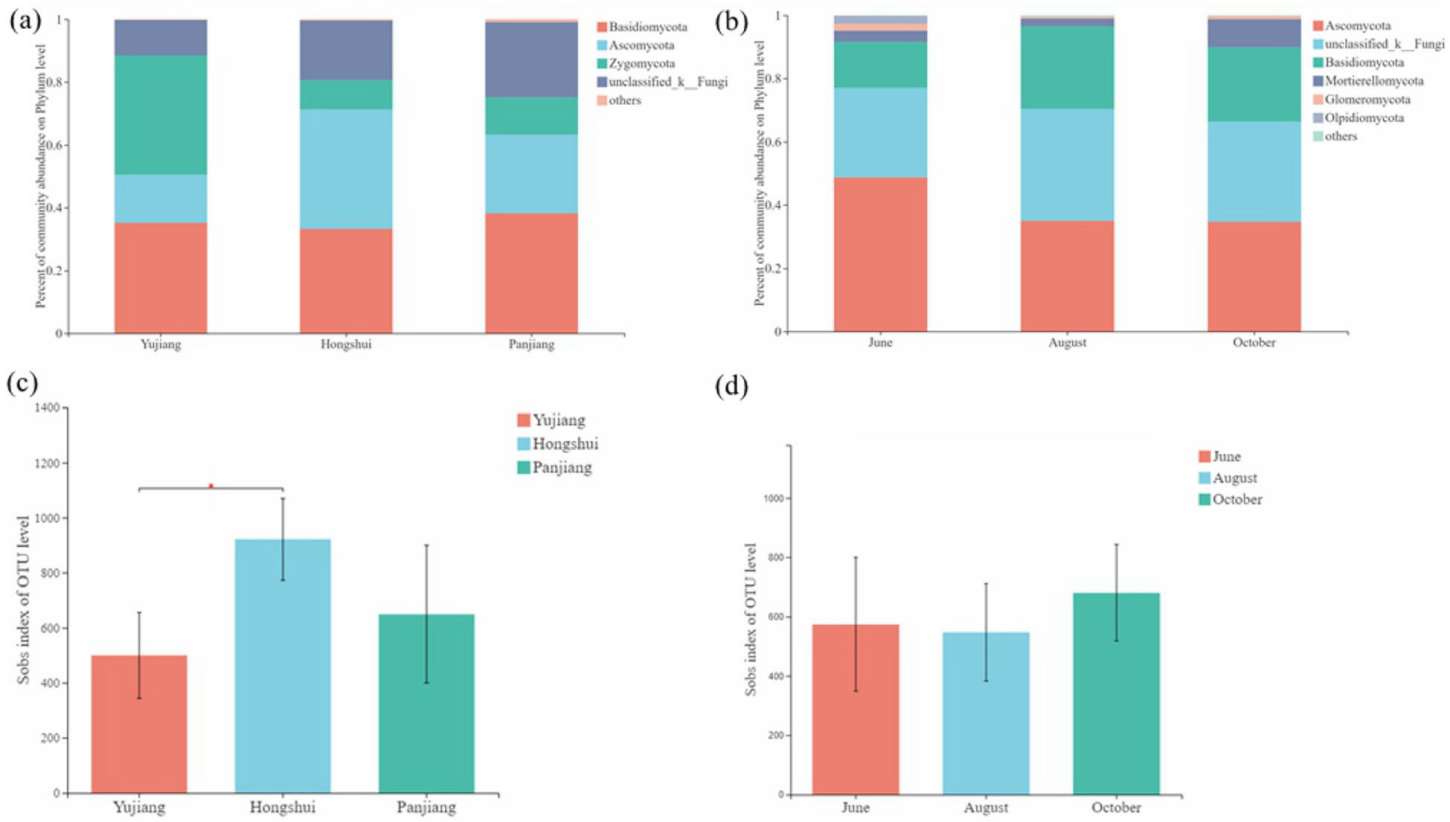
The fungal rhizosphere community during different fruiting periods and small watersheds. **a** relative abundance at the phylum level of **a** small watersheds, and **b** different fruiting period. The boxplot of Shannon–Weiner index of **c** small watersheds and **d** different fruiting periods. **P < 0.01

The fungal communities included *Ascomycota*, *Basidiomycota*, and *Zygomycota* (Fig. 1a). The *Zygomycota* (37.99%) was most abundant in the Yujiang River, followed by *Basidiomycota* (35.14%). The *Ascomycota* (38.11%) and *Basidiomycota* (33.24%) were most abundant in the Hengshui River. The *Basidiomycota* (38.15%) group is the most abundant, followed by *Ascomycota* (25.14%) in the Panjiang River. In different fruiting periods, the *Ascomycota*, *Basidiomycota*, and *Mortierellomycota* were the main groups. *Ascomycota* was the most abundant (48.70% in the young fruit period, 34.96% in the closed immature period, and 34.66% in the mature period), followed by *Basidiomycota* (14.47% in the young fruit period, 26.07% in the closed immature period, 23.43% in the mature period) (Fig. 1b). All samples contained a large number of unidentified fungal groups, and it may be necessary to strengthen the sequencing depth in order to identify them. A total of 2,059,189 sequences were obtained from the soil samples, which were divided into 2112 OTUs. The OTU and Shannon index dilution curves of each sample gradually flattened (Figure S1). The quantity can reflect the vast majority of microbial diversity information in the sample. The student *t*-test showed that the Shannon index of alpha diversity varies significantly between the Yujiang and Hongshui River watersheds, and the diversity was highest in the Hongshui River (*P* < 0.01, Fig. 1c). The Shannon index of different fruiting periods was highest in the mature period (mid-October), but there was no significant difference between periods (Fig. 1d).

### Fungal community variation among sampling compartments

**Fig. 2:**
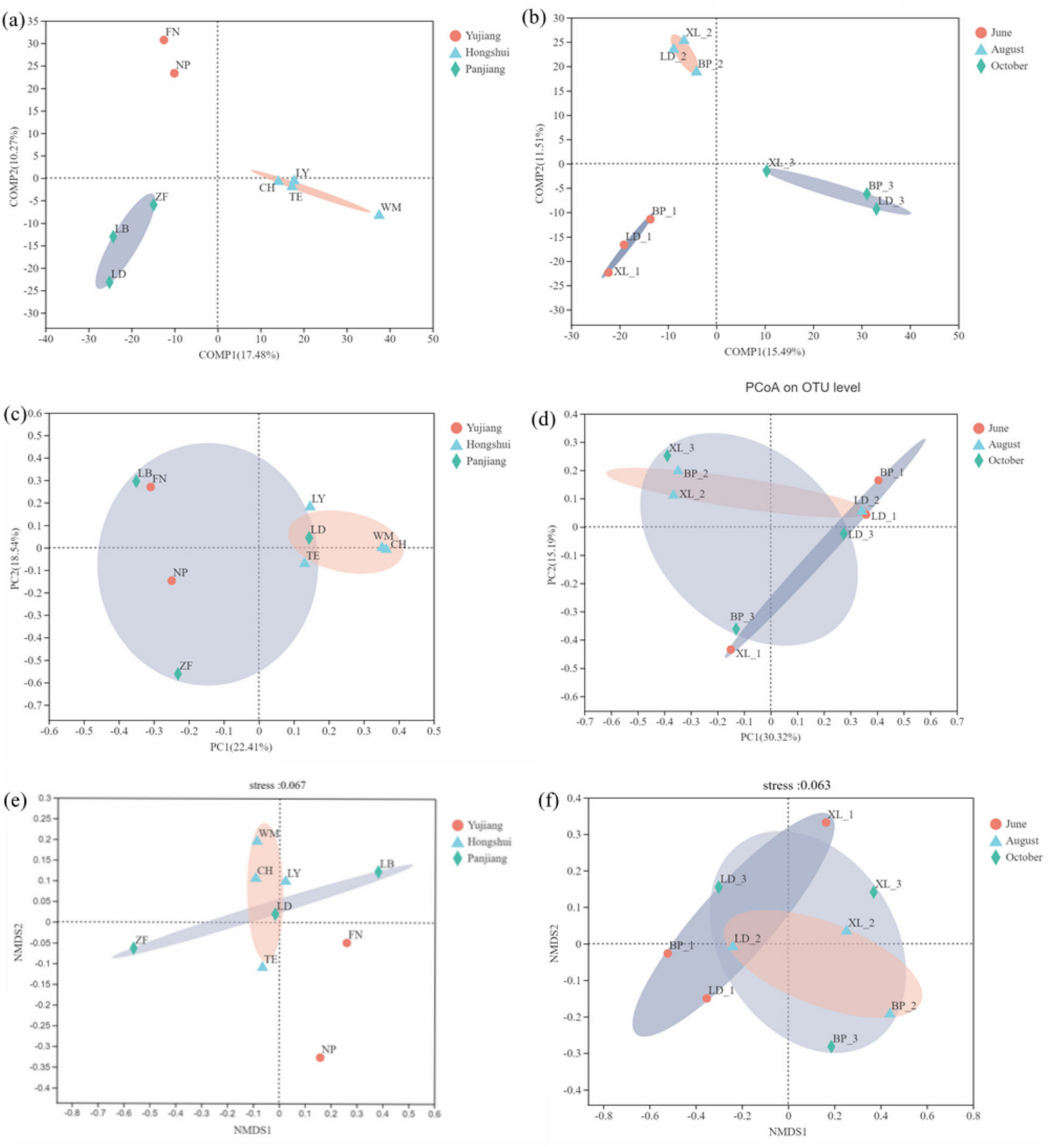
Partial Least Squares Discriminant Analysis (PLS-DA) to classify the objects of study according to the observed or measured values of several variables. The principal coordinates analysis (PCoA) and non-metric multidimensional scaling (NMDS) of all samples using Bray-Curtis distance. **a** PLS-DA small watersheds; **b** PLS-DA fruit period; **c** PCoA small watersheds; **d** PCoA fruit period; **e** NMDS small watersheds; **f** NMDS fruit period. It is generally believed that stress < 0.2 can be represented by 2d NMDS point graph, which has certain explanatory significance. When stress < 0.1, it can be considered a good ranking. When stress < 0.05, it is the most representative

Different watersheds and fruiting periods were used as observation objects to analyze fungal community composition based on OTU levels. The watershed PLS-DA analysis divided the samples from Panjiang, Yujiang, and Hongshui into three categories, demonstrating the feasibility of the watershed as the unit of observation (Fig. 2a). The PLS-DA data also indirectly shows that the watershed may affect the niche differentiation of the fungal community in the rhizosphere of *C. migao*. Community composition was affected by the fruiting period (Fig. 2b). On the Beta diversity scale, PCoA and non-metric multidimensional scaling (NMDS) analysis was performed on the two observation samples using Bray-Curtis distance to evaluate the correlation of fungal community composition. The PCoA analysis of the small watershed (Fig. 2c) scale was highly correlated between the Yujiang and Hongshui River samples, and the Panjiang River watershed was poorly correlated (PC1 22.41%, PC2 18.54%). The correlation between different fruiting periods was poor (Fig. 2d), perhaps due to the large differences between observation points (PC1 37.31%, PC2 16.47%). NMDS analysis showed that the stress of different small watersheds is 0.052 (Fig. 2e), and the ranking results are good. The results show that the observation samples of the Hongshui River Basin are relatively similar and distributed, while the observation groups of the Yujiang River Basin and Panjiang River Basin are more scattered and the distance is larger. According to the results of different fruit period analysis (Fig. 2f), the stress is 0.063, and the ranking result is also good, but the observation points in the three periods are relatively scattered.

### The stability and recruitment of root rhizosphere fungi

**Fig. 3:**
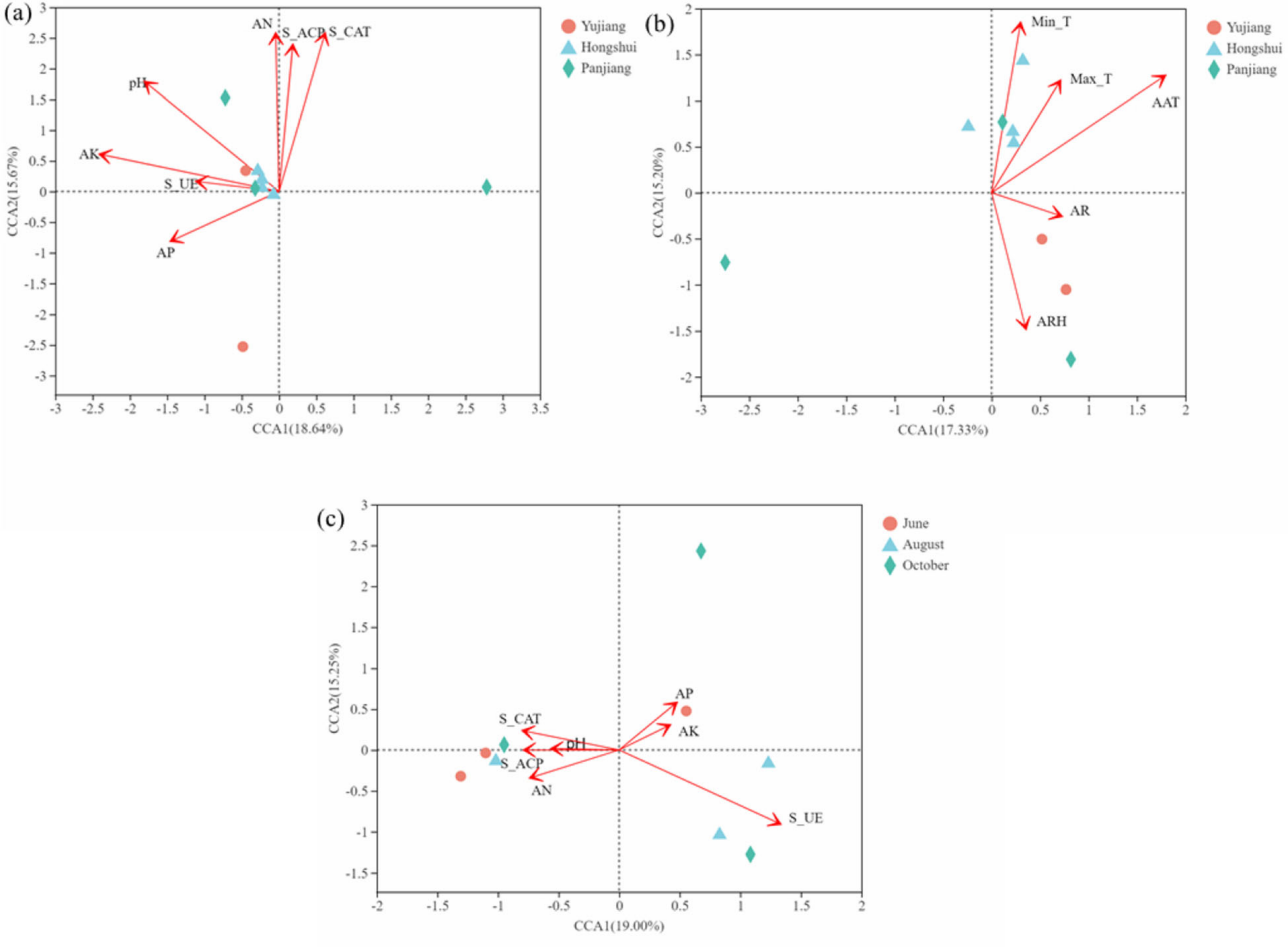
Canonical Correlation Analysis (CCA). **a** CCA, small watersheds of soil; **b** CCA, small watersheds of climate, maximum temperature (Max-T), minimum temperature (Min-T), annual average temperature (AAT), average relative humidity (ARH), annual rainfall (AR); **c** CCA fruit periods. The red arrows represent quantitative environmental factors. Arrow length represents the degree of influence of environmental factors on species data. The distance from the projection point to the origin represents the relative influence of environmental factors on the distribution of the sample community

**Fig. 4:**
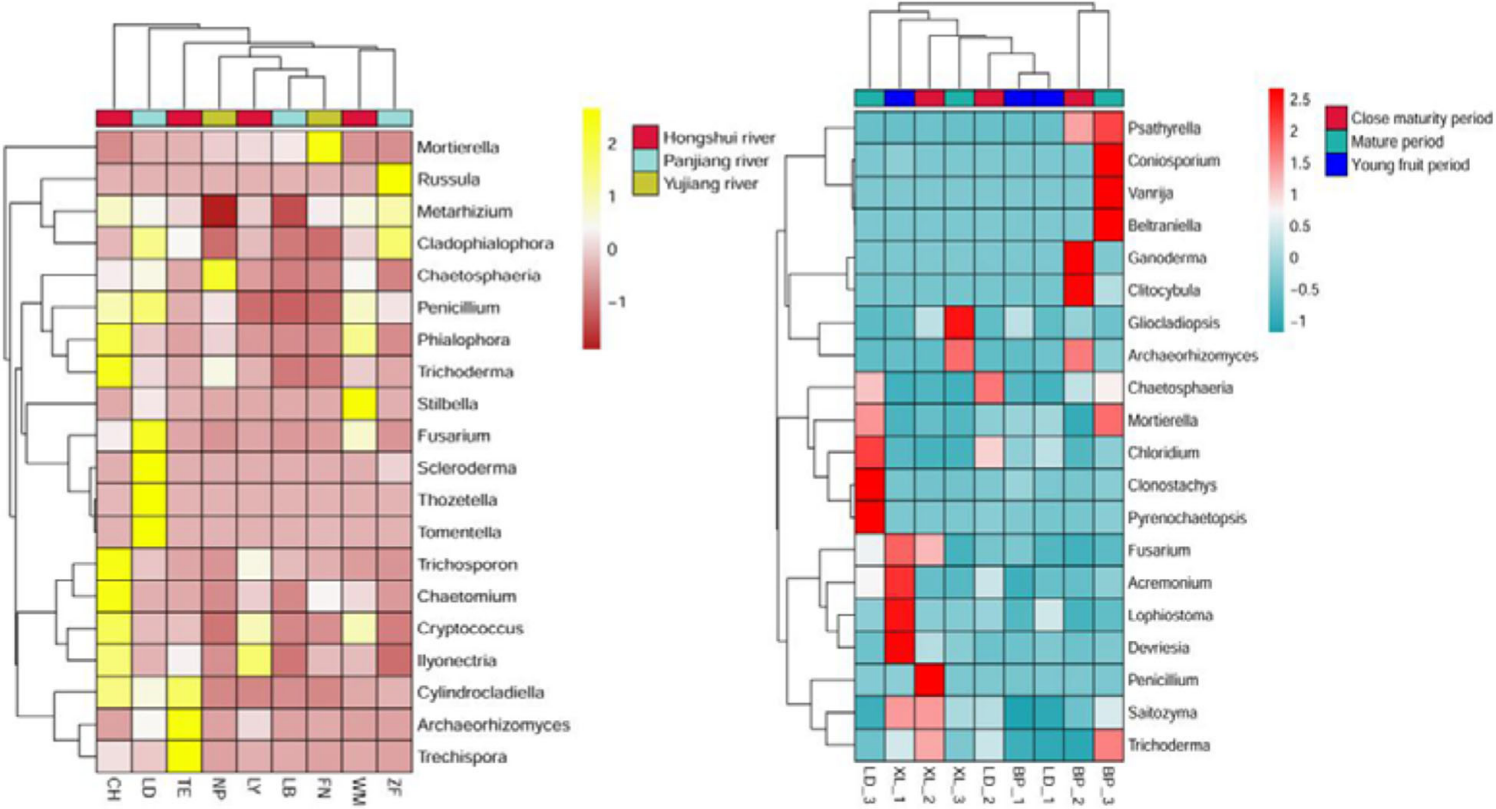
Heatmap of fungal in Rhizosphere Soil of C.migao (genus); **a**, different minor watershed; **b**, different fruit period (exclude “unclassifired” taxa)

Due to the differences between watersheds and fruiting periods, CCA based on the Bray-Curtis distance was conducted to explore the potential relationship between fungal community diversity, soil chemical properties, small watershed climate, and fruiting period. The results of CCA (Fig. 3a) in different small watersheds show that soil K (*r*^2^ = 52.45%, *P* = 0.091) and pH (*r*^2^ = 54.81%, *P* = 0.072) have significant effects on the formation of fungal communities. The influence of the small watershed climate (Table S3) on the rhizosphere fungal community is also greater, among which annual average temperature (AAT) (*r*^2^ = 85.92%, *P* = 0.005) and Min-T (*r*^2^ = 63.68%, *P* = 0.034) are more important for the formation of flora (Fig. 3b). The CCA of different fruiting periods (Fig. 3c) shows that S-UE has a greater influence on the seasonal dynamic change of fungal communities (*r*^2^ = 58.94%, *P* = 0.083). In the class results, the samples of the Hongshui River Basin are well clustered, but the samples of the Panjiang and Yujiang River basins are not clustered (Fig. 4a and Figure S2). Likewise, the samples from three observation sites in different fruit periods did not gather into a single branch in the same season. However, the samples from different observation points are not clustered into one group (Fig. 4b). These data indicate that the fungal community in the rhizosphere of *C. migao* is relatively stable, and the community composition may be mainly due to AK, PH, S-UE, AAT, and other factors.

### Associations between root-associated microbes and environmental variables

**Fig. 5:**
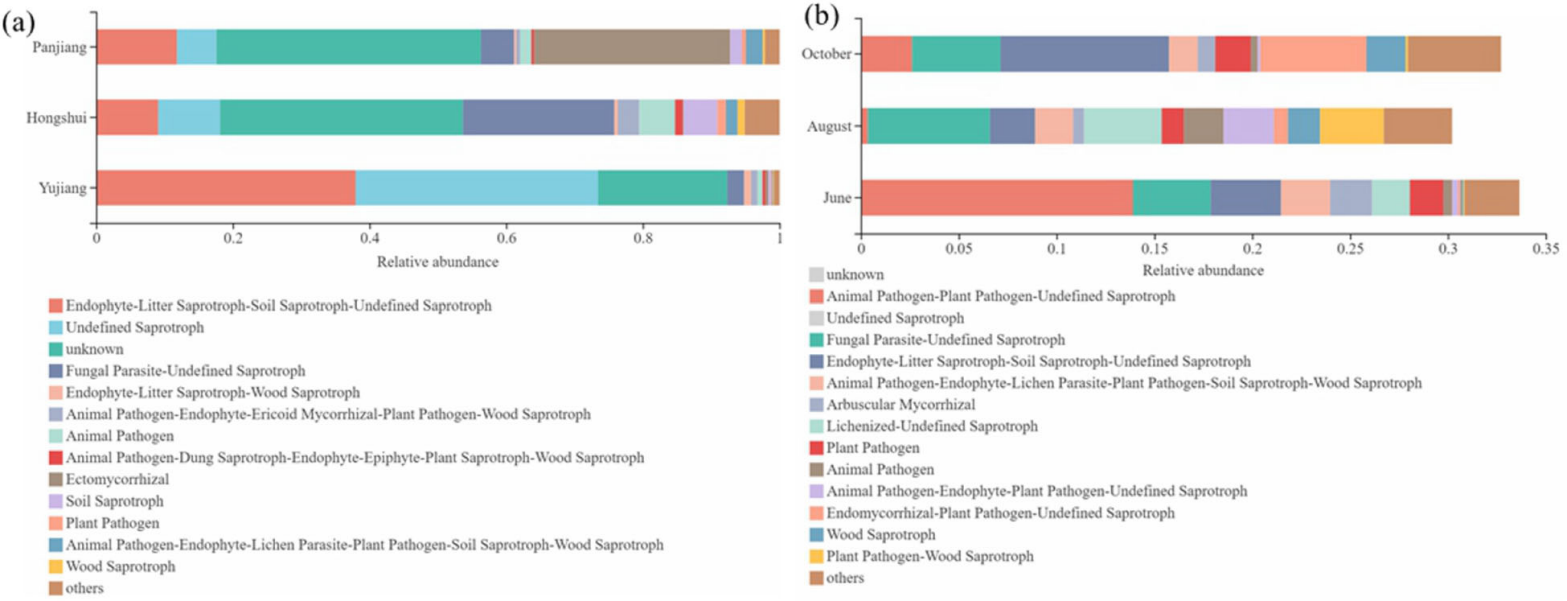
FUNGuild function predicts results. **a** small watersheds; **b** fruiting periods

Why is fungal community composition affected by microclimate and soil properties, and how does this relate to the nutritional type and ecological functions of the fungi? The FUNGuild is a powerful tool for understanding fungal function classification. Functional analysis of different small watersheds shows that the rhizosphere fungi of *C. migao* can be divided into 12 ecological functional groups (Fig. 5a). The functional group of Fungal Parasite-Undefined Saprotroph is the most abundant in the Hongshui River Basin. The functional group Ectomycorrhizal in the Panjiang River Basin is the most abundant, followed by Endophyte-Litter Saprotroph-Soil Saprotroph-Undefined Saprotroph. In the Yujiang River Basin, Endophyte-Litter Saprotroph-Soil Saprotroph-Undefined Saprotroph is the most abundant, followed by Undefined Saprotroph. Rhizosphere fungi can also be divided into 12 ecological functional groups at different fruiting periods (Fig. 5b). In June, the Animal Pathogen-Plant Pathogen-Undefined Saprotroph group is the most abundant, followed by Undefined Saprotroph. In August, the most abundant Undefined Saprotroph group, followed by Fungal Parasite-Undefined Saprotroph. In October, the Undefined Saprotroph group is the most abundant, followed by Endophyte-Litter Saprotroph-Soil Saprotroph-Undefined Saprotroph. Rhizosphere fungi of *C. migao* are predominantly comprised of saprophytic fungi and ectomycorrhizal fungi, perhaps because there are more sludge residues at the contact interface between roots and soil to promote the colonization of fungi with saprophytic functions.

### Relationships between the rhizosphere fungi microbiome and the contents of bioactive components in *C. migao* fruit

We analyzed the relationship between the relevant fungal community and the chemical composition based on Spearman correlation (the top 30% relative genera). In different small watersheds (Figure S2A), α-Terpineol was positively correlated with *Pestalotiopsis*, unclassified Trichocomaceae, *Cladophialophora*, and *Stilbella* (*P* < 0.05), and negatively correlated with *Hypholoma* (P < 0.05). Sabinene was positively correlated with unclassified Thelephoraceae and negatively correlated with *Gibellulopsis*. Crude fat was positively correlated with *Gibellulopsis* and unclassified Ceratobasidiaceae. Sabinene was negatively correlated with unclassified Leotiomycetes. Reducing sugar is positively correlated with unclassified Basidiomycota and negatively correlated with unclassified Venturiales. Total sugar is negatively related to *Cylindrocladiella*. However, soluble polysaccharides and crude polysaccharides were not significantly related to a certain fungus. In different fruiting periods (Figure S2B), total sugar was negatively correlated with *Vanrija,* and α-Terpineol was positively correlated with *Clitocybula*. Crude fat was positively correlated with unclassified Hysteriale, unclassified Sordariomycetes, and unclassified Ceratobasidiaceae. Crude fat was negatively correlated with unclassified Eurotiomycetes. Sabinene was positively correlated with unclassified Hypocreales and unclassified Pleosporales and negatively correlated with *Beltraniella* and unclassified Clavariaceae. Reducing sugar was negatively correlated with unclassified Pleosporales. These results suggest rhizosphere fungi have potential functions in the formation of the chemical components of *C. migao* fruit.

**Fig. 6:**
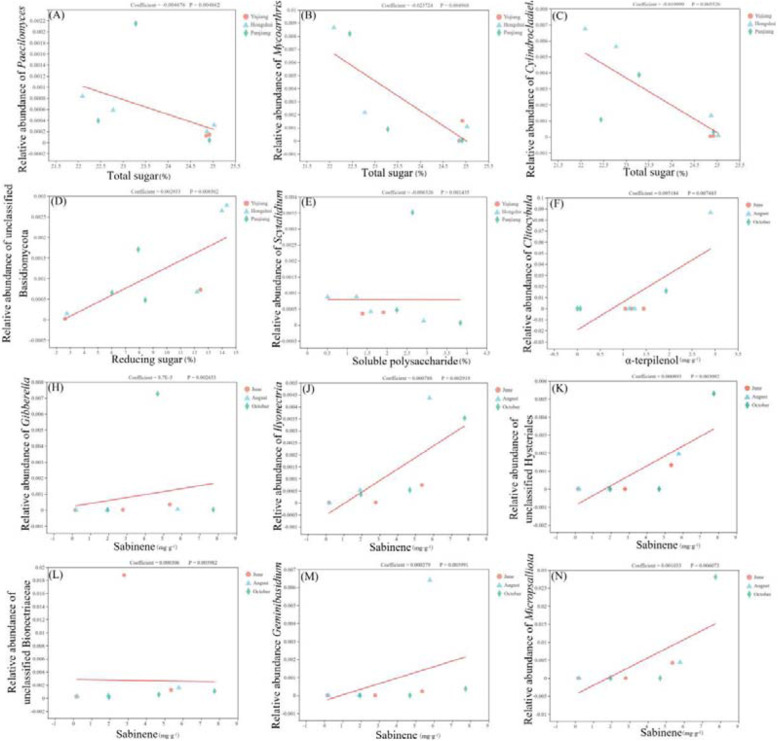
Multivariate Association with Linear Models (MaAslin). **a**-**e**, (small watersheds); **f**-**n**, (Different fruit period); *P* < 0.01

The relationship between fungi and *C. migao* fruit composition variability in small watersheds and fruiting periods was assessed by MaAslin analysis (Fig. 6). Total sugar content gradually decreased with an increasing abundance of *Paecilomyces*, *Mycoarthris*, and *Cylindrocladiella*, and increased with a growing abundance of unclassified Basidiomycota, and soluble polysaccharides decreased with increasing abundance of Scytalidium. The content of α-terpineol increased with an increasing abundance of *Clitocybula,* and sabinene was directly correlated with the abundance of unclassified Hysteriales, unclassified Bionectriaceae, *Gibberella*, *Ilyonectria*, *Micropsalliota*, and *Geminibasidium*.

## Discussion

### The relative of climate and rhizosphere fungi niche

The border of Yunnan, Guizhou, and Guangxi lies in the Guizhou Karma-Fenglin Mountain region. A large number of soluble rocks on the inner surface are exposed, and some insoluble rocks are exposed locally, causing non-karst landforms to be scattered throughout the region. *C. migao* is usually scattered in these non-karst landforms [80]. The transitional folds of the karst outcrop divide these non-karst areas into discontinuous geographical units. In this isolated geographical unit, small watersheds such as the Yujiang, Panjiang, and Hongshui Rivers are formed. This discontinuous geographical distribution pattern forms a unique heterogeneous microenvironment, creating variations in climate between basins at smaller geographic scales and different seasons, profoundly affecting the species distribution in the region [73, 81]. The climate pattern formed in small watersheds also has an important influence on the chemical composition of *C. migao* fruits (Table S3), but the driving force behind this relationship has been unknown. This study shows that the climate differences between small watersheds promote differentiation of the *C. migao* fungal community (Figure S5A). The reason for this may be that there are relatively large differences in soil moisture and temperature between the various watersheds [1] (Tables S1 and S2). Similar effects have been described in *Mussaenda kwangtungensis* interleaf fungus communities, as the *Mussaenda kwangtungensis* populations on islands and the mainland had different niches due to differences in light radiation, which promotes the differentiation of radiation-resistant fungal groups in the two habitats [52]. The difference in rainfall in the upper and lower reaches of the Tarim River in Xinjiang, China, promotes differentiation of related flora and influences plant distribution patterns [8]. Between the edge of the forest and the forest also experience environmental differences, and the fungal biomass and composition also significantly differ in the two small environments [12]. *C.migao* distribution in small watersheds is mainly affected by the southwest monsoon, but due to the influence of karst topography, temperature and rainfall are unevenly distributed [15]. The distribution of *C. migao* in the vertical altitude ranges from about 300 m in Wangmo County, Guizhou Province to 1200 m in Malipo County, Yunnan Province. The river valley region has high perennial temperatures and the air is dry; however, far from the river valley, the temperature gradually decreases, and the humidity increases with the uplift of karst terrain [76], which also becomes an important driving force for the differences of rhizosphere fungal communities. *Boletus edulis* and *Lactarius deliciosus* abound in late autumn and early winter, while *Tuber magnatum* and *Lactarius vinosus* increase in spring. In addition to the life history characteristics of the species, the biggest driving force in fungal community variability is the seasonal changes in soil temperature and humidity [34, 43]. Although there were some fluctuations in the rhizosphere fungi of *C. migao* in different fruit periods (seasons), *Mortierella*, *Saitozyma*, and *Fusarium* with higher abundance in different fruit periods (seasons) did not show obvious changes with fruit periods (season) (Fig. 1b) but were closely related to S-UE and AP. In contrast, other studies have suggested that seasonal changes in soil temperature, humidity, and available water indirectly affect the seasonal dynamics of fungal communities. However, other studies have confirmed that the negative feedback adjustment between some soil pathogens and their hosts is not affected by abiotic environmental conditions such as temperature and soil moisture [25]. Pathogenic fungi accounted for 7.35–12% of the rhizosphere soil population of *C. migao* in different fruiting periods (Fig. 4b). *Mortierella*, *Saitozyma*, and *Fusarium* all have pathogenic characteristics. A large number of pathogenic fungi in the rhizosphere fungi group of *C. migao* explained the reason why the rhizosphere fungi did not show regular changes with seasonal shifts [3, 61, 63]. In general, the unique climate formed in the small watershed greatly promoted the differentiation of the fungal community in the rhizosphere of *C. migao.* It is generally believed that the fungi in the dry-hot valleys, mainly thermophilic fungi, are more sensitive to temperature changes [54]. Although the distribution of *C. migao* is located on both banks of the dry and hot valley, because some populations are distributed at higher altitudes, the environmental temperature is relatively low; many transitional fungi may also be distributed. The response of *C. migao* rhizosphere fungi and fruit chemical components to this small watershed climate provides a perspective that host plants and rhizosphere fungi may have special responses to climate change, potentially changing carbon and nutrient cycles and plant-fungi relationship further affects the chemical composition of the fruit [5].

### Different response of rhizosphere fungal to soil factors

In this study, we not only observed that the climatic differences in the small watershed have a significant impact on the community structure of *C. migao* rhizosphere fungi but also found that the soil chemistry has an important influence on the community structure of *C. migao* rhizosphere fungi (Figure S4). As an important part of the soil food chain, fungi are important decomposers of organic matter such as rhizosphere litter. The composition of organic matter in the rhizosphere affects the composition of the fungal community [33, 36]. CCA (Fig. 3) and Spearman correlation analysis (Figure S4) revealed that pH, S-CAT, AN are closely related to some fungal communities in different watersheds and different fruit periods. The site conditions of all *C. migao* populations are yellow soil acidic soil is suitable for the colonization of acid-loving fungi (Table S1). Studies have shown that the close relationship between mycorrhizal fungi and pH is the dominant factor in fungal community composition and diversity [55, 56]. The analysis results also show that many species of the Chaetothyriales belong to mycorrhizal fungi, which are closely related to pH, thus explaining the close relationship between the rhizosphere fungi of *C.migao *[30]. Soil enzyme activity is closely related to the abundance of fungal populations, such as S-CAT can reduce the damage of peroxide to fungi and significantly related to S-CAT in the rhizosphere of *C. migao*. Many species in the genus *Cladosporium* and *Trichoderma* can produce CAT enzymes to resist the damage caused by peroxides in the environment [6, 64], *Saitozyma* and *Penicillium,* which are also closely related with strong antagonistic effects on peroxides [17]. The existence of these species can effectively reduce the damage of peroxides to the rhizosphere of *C. migao,* and help build a stable rhizosphere micro-ecosystem. The close relationship between both AN and *C. migao* rhizosphere fungi may be related to the availability of nitrogen resources. It is generally believed that when resources are limited, the abundance of fungal populations through plant symbiosis will increase to enhance the use of soil resources. Improve the restriction of resources on plant growth, and the AN-related *Chaetosphaer* of FUNGuild analysis shows that some of the species tend to have a symbiotic relationship with plants. We used Spearman analysis (Table S8) and found that there was a maximum temperature correlation with S-UE, AK, AAT, and S-ACP. Several studies have revealed that changes in climate alter the structure of the fungal community and thus affect the chemical properties of the soil ([41]; Rousk et al. 2009 [75];). The chemical properties also have very important significance [16]. Among the rhizosphere fungi, the more abundant Ascomycetes, Blastomycetes, and Chytridomycetes are saprophytic, decomposing animal and plant residues. This fungus was very important for the material circulation of the rhizosphere of *C. migao* and the improvement of soil chemical properties [16]. The FUNGuild evidence that most fungal groups of *C.migao* rhizosphere are saprophytic, which is very important to promote the mineralization of organic litter and establish a complete feedback loop between soil, fungi, and plants.

### The relationships between the rhizosphere microbes and *C. migao* fruit composition

Beneficial fungi are very important to maintain the healthy growth of plants and improve plant productivity, a concept applied to many agricultural practices [18, 26, 35]. The effects of rhizosphere fungi on plant growth and productivity are direct or indirect. For example, tomato inoculated with fungi could significantly reduce the disease of *Verticillium dahliae* and *Verticillium Alboatrum* and significantly increase the fruit yield [14]; The interaction between *Salvia miltiorrhiza* and rhizosphere fungi can improve the biomass production of *Salvia miltiorrhiza* and affect the metabolic pathway for tanshinone production. *Colletotrichum fioriniae*, an endophytic fungus from *Mahonia fortunei*, produces indole alkaloids similar to the active components in the bark of *M. fortunei* [21]. On the other hand, although some fungal populations are not directly involved in plant growth and function, they can enhance plant adaptation to changes in water and nutrient availability and salinity, thereby regulating photosynthesis and fruit composition [23, 29, 44]. Most studies of the interaction between plants and rhizosphere fungi are focused on crops and herbal medicinal plants; few studies have explored the interaction mechanism of arbor-type medicinal plants [27]. The rhizosphere fungi associated with the chemical components of *C. migao* fruit can be classified into three categories. The first category is the plant pathogens: *Pestalotiopsis* causes grape and olive fruit spoilage [10, 13, 45], and *Ilyonectria* causes root rot in *Persea americana* and *Laurus nobilis*, which belong to the same family as *C. migao* [7, 68], while *Gibberella* causes rhizosphere death in wheat and maize  [59, 46]. This study provides evidence that sabinene is positively correlated with *Ilyonectria* and *Gibberella*, and α-Terpineol is positively correlated with *Pestalotiopsis*. α-Terpineol and sabinene, both volatile oils with strong antibacterial activity, are relatively abundant in the roots and fruits of *C. migao*. The population abundances of these three pathogens increased in the rhizosphere of *C. migao*, exhibiting a positive feedback effect and reducing disease in *C. migao* by synthesizing large amounts of antibacterial α-Terpineol. *Cylindrocladiella* was closely related to root rot, which can cause root rot of avocado, in which the biomass of healthy roots will gradually decrease, and the plant height will be reduced. Our results show that there is negative feedback between population abundance and the total sugar of fruit, perhaps because *Cylindrocladiella* can reduce the plant biomass and thus plant yield [67]. *Scytalidium* causes fruit decay, especially for pitaya [48]. It also has a negative feedback regulation mechanism with reducing sugar, which may be related to its promoting decay function. The second category belongs to the type of plant growth-promoting fungi: *Cladophialophora* has excellent growth-promoting activity and significantly increased plant growth (including bud and root dry weight, chlorophyll content, flower bud germination, and fruit number) [22], which can promote the accumulation of α-Terpineol, and thus also promotes the growth of *C. migao* fruits. *Vanrija* belongs to the basidiomycetes and can decompose soil to produce sugars and D-aspartate oxidase [28]. Although *Vanrija* was identified in the rhizosphere of *C. migao*, it is Jujube (*Ziziphus jujuba*), and *Ficus carica* and *Pistacia vera* are widely distributed on the fruit surface. *Paecilomyces* are believed to have a certain antibacterial effect and can delay spoilage after fruit harvest [20, 58, 60]. The third category belongs to the saprophytic fungi, which secrete sesquiterpenes and typically colonize the litter, which may protect against pathogens in the *C. migao* rhizosphere [2]. *Beltraniella* mainly exists in the deciduous layer, promoting decomposition and producing enzymes to promote fruit rot [65]. However, this type of fungus is not directly related to the chemical components of C. *migao* fruits, but at nutrient return, material circulation, and supply of *C. migao* resources. Most of these fungal groups are closely related to *C. migao* production of carbohydrates, as sugars are the primary metabolites of plant fruits, and their content is often closely related to the availability of environmental resources. Rhizosphere fungi are an important part of rhizosphere microorganisms, interacting with plants in a complex relationship that is especially important for medicinal plants. First, although some fungal groups are unfavorable to plant growth, they also stimulate the plant body to produce secondary metabolites to cope with the adverse environment, and these secondary metabolites are often the medically active ingredients of medicinal plants [57]. Secondly, although a few florae significantly influence the chemical components of *C. migao* fruits, the features of the fruit may be the result of the coordination of many fungal groups, including those that remain unidentified. Some scholars have proposed the concept of a “core microbiome” of multiple key groups rather than a single group [66]. Future work will strengthen the sequencing depth of *C. migao* rhizosphere fungi and explore the core microbiome that affects the chemical components of *C. migao* fruit to reveal the feedback mechanism between rhizosphere fungi and fruit chemical components.

## Conclusions

Here, we investigated the community composition of the rhizosphere and the chemical composition of *C. migao* fruit in different small watersheds and fruiting periods. We explored the relationship between the chemical properties of the rhizosphere soil, the climate of the small watershed, the rhizosphere fungi, and the composition of sugar and crude fat in *C. migao* fruit. There were significant differences in the rhizosphere communities between watersheds, but no significant differences between fruiting periods, indicating that the unique climate of the small watershed promotes the differentiation of the *C. migao* rhizosphere community. There was a close relationship between the community composition of the *C. migao* rhizosphere and S-UE, AP, and AAT. *Clitocybula* may promote the accumulation of α-terpineol in *C. migao* fruit. *Gibberella*, *Ilyonectria*, *Micropsalliota*, and *Geminibasidium* may promote the accumulation of sabinene. In summary, this study reveals the complex network among rhizosphere fungi, small watershed climate, soil chemistry, and plant fruit metabolites, and provides a feasible foundation for a strategy to improve the industrial and pharmacological value of *C. migao* fruit.

## Methods

### Experimental materials

#### Soil samples

**Fig. 7:**
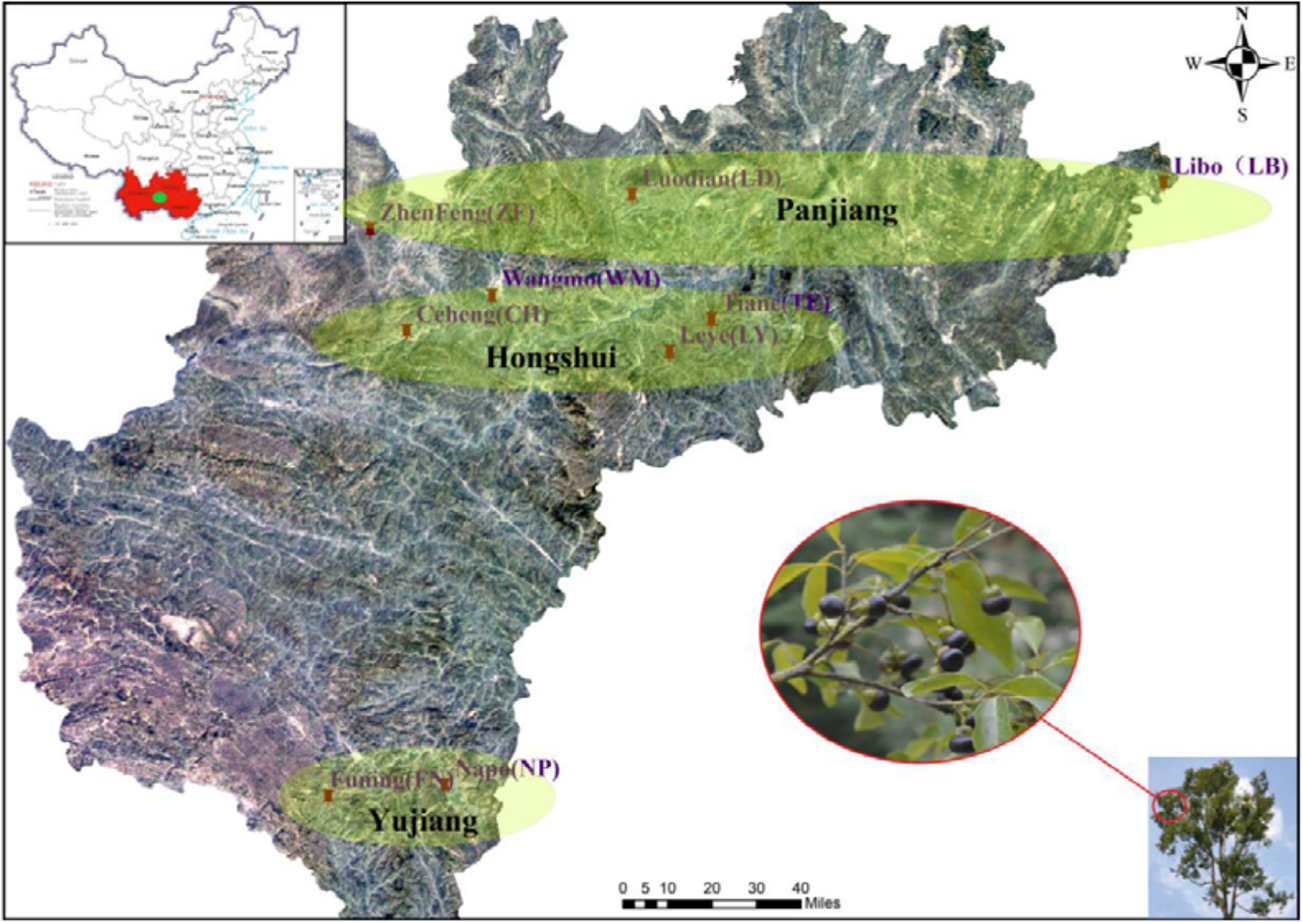
Distribution of *C. migao* sampling points. (The map comes from the standard map service system of the Ministry of natural resources, PRC . The map is authorized for free. http://bzdt.ch.mnr.gov.cn/)

In mid-October 2018, non-disease-carrying *C. migao* plants with a breast diameter of about 32–38 cm were randomly selected five plants from 9 populations of *C. migao* in the watersheds of the Panjiang, Hongshui, and Yujiang rivers. From the three populations of Luodian and Wangmo Counties in Guizhou Province, five plants were randomly selected from each of three growth periods: young fruit (June), closed immature (August), and mature (October). Rhizosphere soil samples were collected around each tree. The humus and topsoil were removed before sampling. A section of 80 cm was vertically excavated along the base of the *C. migao* tree with a sterile shovel to obtain healthy plant roots. Find the fibrous root part along the lateral roots then cut off the branches, shake gently to remove the excess soil, gently shake off the soil within 2 mm of the root system, and then brush the soil still attached to the fibrous roots. After collection, put rhizosphere soil into a sterile plastic, quickly put it in an ice box and bring it back to the laboratory. The soil samples were divided into two parts: one was dried at room temperature (25 °C) for the determination of soil chemical properties, and the other was stored at − 80 °C for genomic extraction of rhizosphere soil fungi. Overall, 90 soil samples were collected, then mix the 5 samples collected from each population evenly (Fig. 7).

#### Fruit samples

Fruit samples were collected from the plants corresponding to the rhizosphere soil of *C. migao*. The fruits were collected from the same small watersheds and fruiting periods, mixed and brought back to the laboratory for drying at low temperature to constant weight. The dried fruit samples were crushed with a grinder and passed through a 40-mesh sieve. All the fruits were collected from the wild because they were not protected species and did not require permission from the Chinese forestry authorities. Overall, 90 fruit samples were collected, then mix the 5 samples collected from each population evenly. All the specimens were identified by Professor Qingwen Sun of Guizhou University of traditional Chinese medicine and preserved in the ecological Laboratory of Guizhou University.

### Experimental method

#### Soil chemistry properties

Analysis of soil properties was performed by conventional methods according to manufacturer protocols, including pH, total nitrogen (TN), total phosphorus (TN), total potassium (TK), alkali hydrolyzed nitrogen (AN), available phosphorus (AP), available potassium (AK) [72], soil acid phosphatase (S-ACP), soil urease (S-UE), and soil catalase (S-CAT) (China, Beijing Solaibao Bioscience Technology). The comparative analysis included analysis of variance (ANOVA) and Tukey *t-*test using SPSS 18.0 (SPSS, Chicago).

#### Extraction of fungal genome

The FastDNA SPINKit for Soil was used to extract fungal genomic DNA according to manufacturer instructions. DNA quality was assessed by 1% agarose gel electrophoresis (5 V·cm^− 1^, 20 min) and UV-1700 spectrophotometry. Amplification primers: ITS1F (CTTGGTCATTTAGAGGAAGTAA) and ITS2R (GCTGCGTTCTTCATCGATGC) were published previously [49]. PCR was performed with TaKaRa rTaq DNA Polymerase in a 20-μl reactio-n containing 10× Buffer (2 μl), 2.5 mM dNTPs (2 μl), Forward and Reverse Primers (5 μM) (0.8 μl), r-Taq Polymerase (0.2 μl), BSA (0.2 μl), and template DNA (10 ng). Cycling conditions: 1 cycle of 95 °C for 3 min, 5 cycles of 95 °C for 30 s, 55 °C for 30 s, and 72 °C 45 s, 72 °C for 10 min, hold at 10 °C (ABI GeneAmp 9700). The PCR products were sequenced using Meiji Biomedical Technology reagents on an Illumina Hiseq platform.

#### Determination of fruit chemical components

The measurement index was selected according to previously published methods. Total sugar, crude polysaccharide, soluble polysaccharide, and reducing sugar were determined by UV-visible spectrophotometry, and crude fat was extracted by the Soxhlet method [40]. HPLC was used to measure α-terpineol and sabinene (Chengdu Desite, purity ≥98%, ThermoFisher Ultimate-3000), using a Hypersil-C18 chromatography column (4.6 mm × 50 mm, 2.6 μm, Thermo Fisher), detection wavelength is 220 nm, column temperature 31 °C, 10 μL injection volume, and 1 ml·min^− 1^ flow rate. Mobile phase: methanol (A)-acetonitrile (B)-0.1% phosphoric acid (C) for gradient elution (elution procedure Table S3). ANOVA and Tukey *t-*test were used to assess differences in fruit chemical composition (SPSS 22.0). The results are shown in Tables S4 and S5.

#### Climate data

Meteorological observation records of Yunnan, Guizhou, and Guizhou for 2018, including temperature and days of sunshine, rainfall, humidity, air pressure, wind speed, and other related data were collected using the inverse-distance weight-interpolation method. This method is commonly used in Acrgis10.2, which is interpolated by the principle of spatial auto correlation [74]. The climate data for each sampling point was extracted from this data (Table S6).

### Data analysis

The sliding window method was used to scan the sequence using Trimmomatic software to eliminate low-quality raw data sequences. When the quality was less than 20 or sequence length less than 50 bp, the sequence was cut.vFlash software was used to splice the qualified double-end raw data. The maximum overlap for sequence splicing was 200 bp, producing the complete paired-end sequence. Split_librarie software in QIIME was used to remove the N bases from the paired-end sequences. Sequences with a single base repeat > 8 were removed, as were sequences < 200 bp to obtain the clean tagged sequence. UCHIME software was used to remove the trim the sequences for operational taxonomic unit (OTU) division; principal coordinate analysis (PCoA), canonical correspondence analysis (CCA), Wilcoxon rank-sum tests, and linear regression based on weighted UniFrac distance (WUF) using the I-Sanger cloud software (http://www.i-sanger.com/).

## Supplementary Information

**Additional file 1 **: **Table S1**. Results of soil chemical properties in different small watershed of *Cinnamonmum migao*. **Table S2**. Results of soil chemical properties in different Different fruit stages of *C. migao*. **Table S3**. Significance of environment factors influencing *C. migao* fruit of different minor watershed. **Table S4**. Elution procedure. **Table S5**. Chemical component content of *C. migao* fruit from different fruit stages. **Table S6**. Chemical component content of *C. migao* fruit from different minor watershed. **Table S7**. Climatic characteristics of the nine collections sites. **Table S8**. Significance of climate factors and soil factor relative. **Figure S1**. The OTU and Shannon index dilution curves of rhizosphere soil fungal community in *C.migao*; Dilution curve is to randomly select a certain number of sequences from the samples, count the alpha diversity index of the corresponding samples of these sequences, draw the curve with the amount of data extracted as the abscissa and the value of alpha diversity index as the ordinate, and judge whether the data amount of this sequencing is sufficient according to whether the curve is smooth or not. **Figure S2**. Heatmap of fungal in Rhizosphere Soil of *C.migao*; (A), (B) Phylum; (C), (D) Class; (E), (F) Order; (G), (H)Family; (I), (J)Species(exclude “unclassifired” taxa). **Figure S3**. Network analysis (two-factor). The red line represents a positive correlation and the green line represents a negative correlation; (A), different small watersheds (B), different fruit periods. **Figure S4**. Kruskal-wallis rank sum test. (a), (different small watersheds); (b), (different fruit periods); *representative the *p*<0.05. **Figure S5**. Network analysis (two-factor). The red line represents a positive correlation and the green line represents a negative correlation; (different small watersheds); (B), (different fruit period); Maximum temperature (Max-T), Minimum temperature (Min-T), Annual average temperature (AAT), Average relative humidity (ARH), Annual rainfall (AR).

## Data Availability

The datasets used or analysed during the current study are available from the corresponding author on reasonable request.

## Abbreviations

Max-T: Maximum temperature
Min-T: Minimum temperature
AAT: Annual average temperature
ARH: Average relative humidity
AR: Annual rainfall

## References

[1] Aurélie V, Odile B, Angélique D, Marina H, Corinne C, Noëlle B, Sophie D, Anne F, Fabien J, Olivier B, Robert D, Philippe NB, Françoise E, Béatrice L (2014). Diversity and spatiotemp-oral dynamics of bacterial communities: physicochemical and other drivers along an acid mine drainage. FEMS Microbiol Ecol.

[2] Ayer WA, Shan R, Trifonov LS, Hutchison LJ (1998). Sesquiterpenes from the nematicidal fu-ngus clitocybula oculus in honour of professor G. H. Neil towers 75th birthday. Phytoche-mistry.

[3] Baldrian P, Kohout P (2017). Interactions of saprotrophic fungi with tree roots: can we observe the emergence of novel ectomycorrhizal fungi?. New Phytol.

[4] Bafana A (2013). Diversity and metabolic potential of culturable root-associated bacteria fro-m *Origanum vulgarein* sub-Himalayan region. World J Microbiol Biotechnol.

[5] Barcelo M, Bodegom VP, Soudzilovskaia NA (2019). Climate drives the spatial distribution of mycorrhizal host plants in terrestrial ecosystems. J Ecol.

[6] Bussink H, Oliver RP (2001). Identification of two highly divergent catalase genes in the fung-al tomato pathogen, *Cladosporium fulvum*. FEBS J.

[7] Cabral A, Rego C, Crous PW, Oliveira H (2012). Virulence and cross-infection potential of *ily-onectri*a spp. to grapevine. Phytopathol Mediterr.

[8] Castano C, Alday JG, Parlade J, Pera J, Aragon JM, Bonet JA (2017). Seasonal dynamics of the ectomycorrhizal fungus Lactarius vinosus are altered by changes in soil moisture and temperature. Soil Biol Biochem.

[9] Chen ML, Zhou T, Jiang WK, Jin YL, Yang ZN (2011). Researches on relationship between genetic differentiation and chemical variation of *Cinnamomum migao*. China J Chinese Mater Med.

[10] Chen T, Lu J, Kang B, Lin M, Ding L, Zhang L, et al. Antifungal activity and actio-n mechanism of ginger oleoresin against pestalotiopsis microspora isolated from Chinese olive fruits. Front Microbiol. 2018. 10.3389/fmicb.2018.02583. 10.3389/fmicb.2018.02583 PMC6218584 30425698

[11] Chowdappa K, Mohan SP, Lakshmi JM, Upreti KK (2013). Growth stimulation and induction of systemic resistance in tomato; against early and late blight by Bacillus subtilis-OTPB1 or *trichoderma*; *harzianum* OTPB3. J Biol Control.

[12] Crockatt ME (2012). Are there edge effects on forest fungi and if so do they matter. Fungal Biol Rev.

[13] Deng JX, Sang H, Hwang YS, Lim BS, Yu SH (2013). Postharvest fruit rot caused by *Pestal-otiopsis* sp. on grape in Korea. Aust Plant Dis Notes.

[14] De Jonge R, Van Esse HP, Maruthachalam K, Bolton MD, Santhanam P, Saber MK, Thomma BP (2012). Tomato immune receptor Ve1 recognizes effector of multiple fungal pathogens uncovered by genome and RNA sequencing. PNAS.

[15] Dong GH, Zheng ZJ, Ma XZ, Huang XZ (2020). Characteristics of low-frequency oscillations in the Hambantota port during the southwest monsoon. Ocean Eng.

[16] Ekman S, Jorgensen PM (2002). Towards a molecular phylogeny for the lichen family Pannari-aceae (Lecanorales, Ascomycota). Botany.

[17] Fraaije MW, Roubroeks HP, Hagen WR, Van Berkel WJ (1996). Purification and Characterizat-ion of an intracellular catalase-peroxidase from *Penicillium Simplicissimum*. FEBS J.

[18] Guevaraavendano E, Bejaranobolivar AA, Kielmartinez A, Ramirezvazquez M, Mendezbravo A, Von Wobeser EA, Reverchon F (2019). Avocado rhizobacteria emit volatile organic com-pounds with antifungal activity against *Fusarium solani*, *Fusarium* sp. associated with Ku-roshio shot hole borer, and *Colletotrichum gloeosporioides*. Microbiol Res.

[19] Guo B, Luo W, Zang WN (2020). Spatial-temporal shifts of ecological vulnerability of Karst Mountain ecosystem-impacts of global change and anthropogenic interference. Sci Total Environ.

[20] Guldur ME, Dikilitas M (2011). Pistachio diseases in the southeastern anatolian region. Acta Hortic.

[21] Hang HW, Gang LI, Xiao PP, Hong XL (2018). Secondary metabolites from colletotrichum fio-riniae f18, an endophytic fungus isolated from the medicinal plant mahonia fortunei. Acta Pharm Sinica.

[22] Harsonowati W, Marian M, Narisawa K. The effectiveness of a dark septate endophytic fungus, cladophialophora chaetospira sk51, to mitigate strawberry fusarium wilt disease a-nd with growth promotion activities. Front Microbiol. 2020. 10.3389/fmicb.2020.00585. 10.3389/fmicb.2020.00585 PMC7174500 32351466

[23] Harman GE (2011). Multifunctional fungal plant symbionts: new tools to enhance plant growth and productivity. New Phytol.

[24] Hartmann A, Rothballer M, Schmid M (2008). Lorenz Hiltner, a pioneer in rhizosphere microb-ial ecology and soil bacteriology research. Plant Soil.

[25] Hawkes CV, Kivlin SN, Rocca JD, Huguet V, Thomsen M, Suttle KB (2011). Fungal commu-nity responses to precipitation. Glob Chang Biol.

[26] Hu D, Zhang S, Baskin JM, Baskin CC, Wang Z, Liu R, Huang Z (2019). Seed mucilage int-eracts with soil microbial community and physiochemical processes to affect seedling eme-rgence on desert sand dunes. Plant Cell Environ.

[27] Huang W, Long C, Lam E (2018). Roles of plant-associated microbiota in traditional herbal m-edicine. Trends Plant Sci.

[28] Imanishi D, Abe K, Kera Y, Takahashi S (2018). Draft genome sequence of the yeast *vanrija humicola* (formerly *Cryptococcus humicola*) strain UJ1, a producer of daspartate oxidas. Genome Announc.

[29] Jaber LR, Enkerli J (2017). Fungal entomopathogens as endophytes: can they promote plant gr-owth?. Biocontrol Sci Tech.

[30] Johannes R, Erland B, Christian LL, Catherine AL, Rob K (2010). Soil bacterial and fungal co-mmunities across a pH gradient in an arable soil. ISME.

[31] Jones DL, Nguyen C, Finlay RD (2009). Carbon flow in the rhizosphere: carbon trading at the soil-root interface. Plant Soil.

[32] Köberl M, Schmidt R, Ramadan EM, Bauer R, Berg G (2013). The microbiome of medicinal plants: diversity and importance for plant growth, quality, and health. Front Microbiol.

[33] Laurent P, Jos MR, Philippe L, Wim HV (2013). Going back to the roots: the microbial ecology of the rhizosphere. Nat Rev Microbiol.

[34] La V, Agueda B, Agreda T, Martinezpen F, Parlade J, Pera J (2013). Seasonal dynamics of *Boletus edulis* and *Lactarius deliciosus* extraradical mycelium in pine forests of central Sp-ain. Mycorrhiza.

[35] Lee Y, Han JH, Kang BR, Kim YC (2019). Dibutyl succinate, produced by an insect-pathogen-ic fungus, *Isaria javanica* pf185, is a metabolite that controls of aphids and a fungal dise-ase, anthracnose. Pest Manag Sci.

[36] Lebeis SL, Paredes SH, Lundberg DS, Breakfield NW, Gehring J, Mcdonald M, Tijana GD, Corbin DJ, Susannah GT, Jeffery LD (2015). Salicylic acid modulates colonization of the root microbiome by specific bacterial taxa. Science.

[37] Li Y, Wang S, Lu M, Zhang Z, Chen M, Li S, Cao R (2019). Rhizosphere interactions betwe-en earthworms and arbuscular mycorrhizal fungi increase nutrient availability and plant gr-owth in the desertification soils. Soil Tillage Res.

[38] Li YH, Yang X. Protective effect of *Cinnamomum migao* in Guizhou on acute Myoca-rdial ischemia injury. Info Tradit Chin Med. 2020;37(03):4–8.10.19656.

[39] Li XW, Li J, Henk W (2008). Flora of China.

[40] Liu G, Liu YC, Kang XJ (2017). Content determination of total flavonoids and sugar comp-onents in *Cinnamomum migao*. J Agric Sci.

[41] Liu C, Ding N, Fu Q, Brookes PC, Xu J, Guo B, Li N (2016). The influence of soil proper-ties on the size and structure of bacterial and fungal communities along a paddy soil chr-onosequence. Eur J Soil Biol.

[42] Lu LM, Mao LF, Yang T, Ye JF, Liu B, Li HL, Sun M, Joseph TM, Sarah M, Hu HH, Niu YT, Dan XP, Chen YH, Stephen AS, Chen M, Xiang KL, Le CT, Viet CD, An ML, Pamela SS, Douglas ES, Li JH, Chen ZD (2018). Evolutionary history of the angiosperm flora of China. Nat.

[43] Marjanovic Ž, Glisic A, Mutavdžic D, Saljnikov E, Bragato G (2015). Ecosystems supporting tuber magnatum Pico production in Serbia experience specific soil environment seasonality that may facilitate truffle lifecycle completion. Appl Soil Ecol.

[44] Mayerhofer MS, Kernaghan G, Harper KA (2013). The effects of fungal root endophytes on p-lant growth: a meta-analysis. Mycorrhiza.

[45] Maharachchikumbura SS, Guo L, Chukeatirote E, Bahkali AH, Hyde KD (2011). Pestalotiopsismorphology, phylogeny, biochemistry and diversity. Fungal Divers.

[46] Miller J D . Factors that affect the occurrence of fumonisin.[J]. Environ Health Perspect. 2001;109(Suppl 2):321–4. 10.1289/ehp.01109s2321 PMC1240682 11359702

[47] Ministry of Ecology and Environment, PRC (2011). The list of red species of biodiversity in China: higher plants volume.

[48] Mohd MH, Salleh B, Zakaria L (2013). Identification and molecular characterizations of Neosc-ytalidium dimidiatum causing stem canker of red-fleshed dragon fruit (Hylocereus polyrhiz-us) in Malaysia. J Phytopathol.

[49] Mukherjee PK , Chandra J, Retuerto M, et al. Oral Mycobiome Analysis of HIV-Infected Patients: Identification of Pichia as an Antagonist of Opportunistic Fungi[J]. PLoS Pathog. 2014;10(3):e1003996. 10.1371/journal.ppat.1003996. 10.1371/journal.ppat.1003996 PMC3953492 24626467

[50] Petr C, Stefano M, Eva K, Birgit W, Kateřina D, Jiři B, Jorg S, Christina B, Pertti JM, Ricardo J, Georg G, Norman G, Gustaf H, Juri P, Robert M, Olga S, Tim U, Christa S, Andreas R, Ha-na S (2018). A plant-microbe interaction framework explaining nutrient effects on primary production. Nat Ecol Evol.

[51] Perezjaramillo JE, Carrion VJ, Hollander MD, Raaijmakers JM (2018). The wild side of plant microbiomes. Microbiome.

[52] Qian X, Li S, Wu B, Wang Y, Ji N, Yao H (2020). Mainland and island populations of *Mus-saenda kwangtungensis* differ in their phyllosphere fungal community composition and network structure. Sci Rep.

[53] Rilling J, Acuña J, Nannipieri P, Cassan F, Maruyama F, Jorquera M (2018). Current opinion and perspectives on the methods for tracking and monitoring plant growth-promoting bact-eria. Soil Biol Biochem.

[54] Rudgers JA, Belldereske L, Crawford KM, Emery SM (2015). Fungal symbiont effects on dun-e plant diversity depend on precipitation. J Ecol.

[55] Rousk J, Brookes PC, Baath E (2010). Investigating the mechanisms for the opposing pH relat-ionships of fungal and bacterial growth in soil. Soil Biol Biochem.

[56] Johannes R, Philip CB, Erland B (2009). Contrasting soil pH effects on fungal and bacterial growth suggest functional redundancy in carbon mineralization. Appl Environ Microbiol.

[57] Rosa E, Woestmann L, Biere A, Saastamoinen M (2018). A plant pathogen modulates the effe-cts of secondary metabolites on the performance and immune function of an insect herbiv-ore. Oikos.

[58] Sabbarao KV, Michailides TJ, Morgan DP (1993). Effects of osmotic potential and temperature on growth of two pathogens of figs and a biocontrol agent. Phyto Pathol.

[59] Schroeder HW, Christensen JJ, Christensen JD, Platzchristensen JJ, Schroeder H (1963). Factors affecting resistance of wheat to scab caused by Gibberella zeae. Phytopathology.

[60] Singh YP (1998). Preharvest mycobial population of Indian jujube fruits (*Ziziphus mauritiana* Lamk.) and their implications in postharvest pathogenesis. Mycopathologia.

[61] Sterkenburg E, Bahr A, Durling MB, Clemmensen KE, Lindahl BD (2015). Changes in fungal communities along a boreal forest soil fertility gradient. New Phytol.

[62] Taylor DL, Hollingsworth TN, Mcfarland JW, Lennon N, Nusbaum C, Ruess RW (2014). A fi-rst comprehensive census of fungi in soil reveals both hyperdiversity and fine-scale ni-che partitioning. Ecol Monogr.

[63] Tedersoo L, Bahram M, Põlme S, Anslan S, Riit T, Kõljalg U, Nilsson RH, Hildebrand F, Abarenkov K. Response to Comment on “Global diversity and geography of soil fungi”. Science. 2015;349(6251). 10.1126/science.aaa5594. 10.1126/science.aaa5594 26315429

[64] Teder T, Boeglin WE, Schneider C, Brash AR (2017). A fungal catalase reacts selectively with the 13S fatty acid hydroperoxide products of the adjacent lipoxygenase gene and exhibits 13S-hydroperoxide-dependent peroxidase activity. Biochim Biophys Acta.

[65] Tziros GT, Lagopodi AL, Tzavellaklonari K (2008). Alternaria alternata fruit rot of pomegranat-e (*Punica granatum*) in Greece. Plant Pathol.

[66] Toju H, Peay KG, Yamamichi M, Narisawa K, Hirum K, Naito K, Kiers ET (2018). Core mi-crobiomes for sustainable agroecosystems. Nat Plants.

[67] Urbeztorres JR, Haag P, Bowen P, Ogorman DT (2014). Grapevine trunk diseases in British Columbia: incidence and characterization of the fungal pathogens associated with esca and petri diseases of grapevine. Plant Dis.

[68] Vitale A, Aiello D, Guarnaccia V, Perrone G, Stea G, Polizzi G (2012). First report of root rot caused by Ilyonectria (=Neonectria) macrodidyma on avocado (Persea americana) in Italy. J Phytopathol.

[69] Wang JH, Zheng N, Wang J (2016). Main nutritional components in *Cinnamomum migao* see-d. Guizhou Agric Sci.

[70] Wu HM, Qin XJ, Wang JY, Wu LK, Chen J, Fan JK, Zheng L, Haipeng T, Yasir A, Lin W, Luo XM, Lin S, Lin WX (2019). Rhizosphere responses to envi-ronmental conditions in Radix *pseudostellariae* under continuous monoculture regimes. Agric Ecosyst Environ.

[71] Wu BL, Wang FQ, Tang Y, Hu YS, Liu Y, Liu WQ (2019). Therapeutic effect of oil of *Cinnamomum migao* on atrial fibrillation in rats. Lishizhen Med Mater Med Res.

[72] Zhu XF, Chen HS. [runoff and nitrogen loss characteristics in soil-epikarst system on a karst shrub hillslope]. J Applied Ecology. 2017. 10.13287/j.1001-9332.201707.029. 10.13287/j.1001-9332.201707.029 29741050

[73] Xu Y, Wang S, Bai X, Shu D, Tian Y. Runoff response to climate change and human activities in a typical karst watershed, SW China. PLOS ONE. 2018;13(3). 10.1371/journal.pone.0193073. 10.1371/journal.pone.0193073 PMC5832221 29494602

[74] Yang WJ, Zhao Y, Wang D, Wu HH, Lin AJ, He L. Using principal components Ana-lysis and IDW interpolation to determine spatial and temporal changes of surface water quality of Xin'anjiang river in Huangshan. China. Int J Environ Res Public Health. 2020.17(8). 10.3390/ijerph17082942. 10.3390/ijerph17082942 PMC7215294 32344554

[75] Yu C, Han F, Fu G (2019). Effects of 7 years experimental warming on soil bacterial and fu-ngal community structure in the Northern Tibet alpine meadow at three elevations. Sci Total Environ.

[76] Zhao S, Li HY, Liu N, Qiu DW, Liu ZR (1990). Studies on the original plants, congeneric v-arieties and easily mixed varieties of *Cinnamomum migao*. J G Col Trad Chin Med.

[77] Zeng T, William AA, Brandy M (2013). Microscale characterization of sulfur speciation in L-ake sediments. Environ Sci Technol.

[78] Zhang YP, Qiu DW, Zheng YY, Wu YT, Qin AM (2003). GC-MS analysis of volatile oil fro-m *Cinnamomum migao* of fruit. China J Trad Chin Med Pharm.

[79] Zhang XB, Zhou T, Guo LP, Huang LQ, Jiang WK, Yang ZN, Ma CY (2011). Volatile oil c-ontents correlate with geographical distribution patterns of the Miao ethnic herb Fructus C-innamomi. Acta Ecol Sin.

[80] Zhang PQ, Sun J, Liu XH, Zuo TH (2013). Features of fluvial landform and crust deformatio-ns along the nanpan jiang river-hongshuihe river (middle segment). Quat Sci.

[81] Ze H, Wei S (2019). Spatiotemporal variations in cropland abandonment in the Guizhou-Guangxi karst mountain area. China J Clean Prod.

