# Supplementary material for: Effects of rhizosphere fungi on the chemical composition of fruits of the medicinal plant Cinnamomum migao endemic to southwestern China
Source: BMC Microbiol. 2021 Jul 6;21:206. doi: 10.1186/s12866-021-02216-z (PMC8259389; doi:10.1186/s12866-021-02216-z)
Supplement: Supplementary file 1 — Additional file 1 : Table S1. Results of soil chemical properties in different small watershed of Cinnamonmum migao. Table S2. Results of soil chemical properties in different Different fruit stages of C. migao. Table S3. Significance of environment factors influencing C. migao fruit of different minor watershed. Table S4. Elution procedure. Table S5. Chemical component content of C. migao fruit from different fruit stages. Table S6. Chemical component content of C. migao fruit from different minor watershed. Table S7. Climatic characteristics of the nine collections sites. Table S8. Significance of climate factors and soil factor relative. Figure S1. The OTU and Shannon index dilution curves of rhizosphere soil fungal community in C.migao; Dilution curve is to randomly select a certain number of sequences from the samples, count the alpha diversity index of the corresponding samples of these sequences, draw the curve with the amount of data extracted as the abscissa and the value of alpha diversity index as the ordinate, and judge whether the data amount of this sequencing is sufficient according to whether the curve is smooth or not. Figure S2. Heatmap of fungal in Rhizosphere Soil of C.migao; (A), (B) Phylum; (C), (D) Class; (E), (F) Order; (G), (H)Family; (I), (J)Species(exclude “unclassifired” taxa). Figure S3. Network analysis (two-factor). The red line represents a positive correlation and the green line represents a negative correlation; (A), different small watersheds (B), different fruit periods. Figure S4. Kruskal-wallis rank sum test. (a), (different small watersheds); (b), (different fruit periods); *representative the p<0.05. Figure S5. Network analysis (two-factor). The red line represents a positive correlation and the green line represents a negative correlation; (different small watersheds); (B), (different fruit period); Maximum temperature (Max-T), Minimum temperature (Min-T), Annual average temperature (AAT), Average relative humidity (ARH), Ann [file 12866_2021_2216_MOESM1_ESM.doc]

### Effects of rhizosphere fungi on the chemical composition of fruits of the medicinal plant *Cinnamomum migao* endemic to southwestern China

**journal name: Plant and soli**

Jingzhong Chen^a^, Xiaolong Huang^a^, Bingli Tong^a^, Deng Wang^a^, Jiming Liu^a*^, Xiaofeng Liao^b^, Qinwen Sun^c^

^a^College of Forestry, Guizhou University, Guiyang, 550025, China

^b^Guizhou province Institute of Mountain Resources, Guiyang, 550025, China

^c^College of Pharmacy, Guizhou University of Traditional Chinese Medicine, Guiyang, 550025, China

*corresponding author：[Karst0623@163.com;](mailto:Karst0623@163.com;) Huaxi District, Guiyang City, Guizhou Province, China

Table S1. Results of soil chemical properties in different small watershed of *Cinnamonmum migao*

| **Minor**  **watershed** | Sanple | pH | Total nitrogen TN(g·kg^-1^) | Total potassium TK(g·kg^-1^) | Total phosphorus TP(g·kg^-1^) | Alkali hydrolyzed nitrogen AN(mg·kg^-1^) | Available potassium AK(mg·kg^-1^) | Available phosphorus AP(mg·kg^-1^) | Soil urease S-UE(μg NH3-N·g ^-1^·d^-1^) | Soil acid phosphatase S-ACP(μmol·d^-1^·g^-1^) | Soil catalase S-CAT(ml·g^-1^·20min) |
| --- | --- | --- | --- | --- | --- | --- | --- | --- | --- | --- | --- |
| **Hongshui**  **river** | WM | 5.82±0.19a | 1.82±0.11a | 32.70±0.31a | 0.175±0.00cde | 166.83±15.17d | 819.70±0.30a | 6.06±0.14a | 259.05±21.82ab | 18.15±0.09bc | 0.95±0.01abc |
|  | CH | 4.94±0.02b | 2.15±0.05a | 31.27±0.47ac | 0.178±0.00cd | 250.83±7.65be | 320.37±3.43c | 3.31±0.93c | 129.05±23.75ab | 23.39±0.22c | 0.98±0.01e |
|  | TE | 4.80±0.01ge | 2.24±0.28a | 28.80±1.36c | 0.140±0.01f | 252.00±4.04be | 803.37±6.56a | 4.31±0.01bc | 72.38±9.19a | 15.75±0.63a | 0.92±0.01bf |
|  | LY | 6.82±0.01c | 2.47±0.08a | 37.50±0.29b | 0.153±0.00def | 268.33±10.17b | 818.80±1.08a | 4.97±0.11ab | 135.24±21.82ab | 13.65±0.2d | 0.33±0.03d |
| **Yujiang**  **river** | FN | 5.72±0.03a | 2.61±0.25a | 18.83±0.23e | 0.205±0.01b | 246.17±9.11be | 817.60±0.30a | 4.48±0.10bc | 41.43±1.43a | 16.85±0.41b | 0.88±0.01dbf |
|  | NP | 4.56±0.15e | 2.24±0.32a | 30.17±1.34c | 0.145±0.01f | 166.83±8.41d | 654.33±11.62d | 5.49±0.06ab | 392.38±60ab | 18.94±2.7e | 0.62±0.01af |
| **Panjiang**  **river** | LB | 6.11±0.01d | 2.73±0.19a | 36.00±0.30b | 0.192±0.01bc | 325.5±2.02a | 816.70±0.30a | 4.70±0.04bc | 30.47±8.25b | 18.71±0.32C | 0.9±0.01b |
|  | LD | 4.84±0.02ge | 2.29±0.25a | 8.07±0.15f | 0.265±0.01a | 226.33±7.10e | 816.07±0.83a | 3.34±0.12c | 73.33±8.25a | 20.56±0.65ab | 0.47±0.02abf |
|  | ZF | 4.19±0.01f | 1.73±0.19a | 23.90±0.46d | 0.150±0.01ef | 245.00±6.06be | 259.67±8.17d | 3.41±0.03c | 30.48±8.6a | 13.64±0.03a | 0.88±0d |

In one-way ANOVA of all samples, Turkey was used to compare the multiple means (P < 0.05), which was significant difference; there was no significant difference when the same letter was used, and there was significant difference between different letters. The same below.

Tab. S2 Results of soil chemical properties in different Different fruit stages of *C. migao*

| **fruit stages** | Sanple | pH | Total nitrogen TN(g·kg^-1^) | Total potassium TK(g·kg^-1^) | Total phosphorus TP(g·kg^-1^) | Alkali hydrolyzed nitrogen AN(mg·kg^-1^) | Available potassium AK(mg·kg^-1^) | Available phosphorus AP(mg·kg^-1^) | Soil urease S-UE(μg NH3-N·g ^-1^·d^-1^) | Soil acid phosphatase S-ACP(μmol·d^-1^·g^-1^) | Soil catalase S-CAT(ml·g^-1^·20  min) |
| --- | --- | --- | --- | --- | --- | --- | --- | --- | --- | --- | --- |
| **young fruit**  **period(June)** | BP-1 | 4.68±0.02+a | 0.81±0.08a | 1.49±0.23a | 0.33±0.01a | 233.33±40.41a | 794.92±32.46a | 1.13±0.12a | 481.48±12.83abc | 37.51±0.53ab | 8.58±0.51a |
|  | LD-1 | 6.02±0.05 d | 0.67±0.14a | 0.68±0.07b | 0.27±0.01e | 373.33±40.41c | 63.79±2.34b | 1.21±0.15a | 303.71±84.13ad | 41.02±0.81bc | 3.5±0.72b |
|  | XL-1 | 4.91±0.17a | 0.67±0.01a | 1.41±0.24a | 0.22±0.03d | 256.67±40.41a | 450.31±61.3ce | 1.33±0.12a | 688.92±38.49c | 31.78±1.56a | 0.2±0.02c |
| **Close to maturity**  **[period](../../../../C:/Users/%25E9%2599%2588%25E6%2595%25AC%25E5%25BF%25A0/AppData/Local/youdao/dict/Application/8.9.3.0/resultui/html/index.html#/javascript:;) (August)** | BP-2 | 5.58±0.042 b | 0.14±0.05b | 3.32±0.01de | 0.25±0.03c | 70.01±0b | 403.71±5.68e | 2.52±0.02b | 420.11±60.08bd | 27.58±3.38cd | 0.57±0.01c |
|  | LD-2 | 6.53±0.0465ce | 0.14±0.01A | 2.84±0.08de | 0.21±0.01d | 81.67±20.21b | 34.09±2.69bd | 2.84±0.07b | 380.08±60.11bd | 22.1±0.43d | 0.62±0.01c |
|  | XL-2 | 6.87±0.028c | 0.21±0.01c | 2.77±0.51cde | 0.44±0.03b | 256.67±40.41a | 509.74±2.37c | 3.41±0.08e | 700.08±140.82c | 28.92±0.57acd | 0.45±0.09c |
| **[mature](../../../../C:/Users/%25E9%2599%2588%25E6%2595%25AC%25E5%25BF%25A0/AppData/Local/youdao/dict/Application/8.9.3.0/resultui/html/index.html#/javascript:;) [period](../../../../C:/Users/%25E9%2599%2588%25E6%2595%25AC%25E5%25BF%25A0/AppData/Local/youdao/dict/Application/8.9.3.0/resultui/html/index.html#/javascript:;)**  **(October)** | BP-3 | 5.66±0.03b | 0.16±0.03A | 2.45±0.03c | 0.03±0.01b | 98.15±24.25b | 459.48±45.32ce | 8.58±0.23f | 428.93±44.01bd | 29.66±0.64acd | 6.04±0.29d |
|  | LD-3 | 7.08±0.46e | 0.33±0.03c | 2.26±0.08c | 0.01±0.01b | 135.33±32.33b | 38.93±1.08bd | 9.28±0.31c | 335.25±26.29bd | 30.02±1.04acd | 8.53±1.25a |
|  | XL-3 | 4.57±0.09a | 0.13±0.02b | 2.31±0.07c | 0.02±0.01b | 105.31±12.12b | 125.46±0.3b | 7.64±0.15d | 833.27±85.96c | 29.85±0.87acd | 6.61±0.58d |

Tab. S3 Significance of environment factors influencing *C. migao* fruit of different minor watershed

| **Factor** | S-CAT | S-UE | S-ACP | pH | AK | AK | AP | Maximum temperature | Minimum temperature | Annual average temperature | Average relative humidity | Annual rainfall |
| --- | --- | --- | --- | --- | --- | --- | --- | --- | --- | --- | --- | --- |
| **α_Terpineol(mg·g^-1^）** | -0.42 | -0.23 | 0.13 | -0.08 | 0.17 | 0.53* | 0.07 | 0.31 | 0.00 | -0.16 | -0.23 | -0.09 |
| **Sabinene(mg·g^-1^）** | -0.53* | -0.55* | 0.22 | -0.13 | -0.06 | 0.45* | -0.26 | 0.39 | 0.02 | 0.03 | -0.23 | -0.08 |
| **Total sugar(%)** | 0.39 | 0.31 | 0.00 | 0.35 | 0.22 | -0.15 | 0.42 | -0.29 | -0.24 | -0.31 | 0.12 | -0.16 |
| **Reducing sugar(%)** | 0.08 | 0.03 | 0.23 | -0.05 | 0.65* | -0.07 | 0.22 | -0.61* | -0.63* | -0.17 | -0.11 | -0.12 |
| **Soluble polysaccharide(%)** | -0.03 | -0.02 | -0.36 | 0.09 | -0.45* | 0.06 | 0.07 | 0.40 | 0.42 | 0.23 | 0.15 | -0.22 |
| **Crude polysaccharide(%)** | 0.16 | 0.05 | 0.09 | 0.22 | 0.11 | 0.21 | 0.23 | 0.45* | 0.21 | 0.20 | 0.18 | -0.05 |
| **Crude fat(%)** | 0.05 | 0.01 | -0.56* | 0.08 | -0.11 | -0.39 | 0.07 | -0.58* | -0.39 | -0.69* | 0.21 | 0.09 |

* represents a significant correlation

**Tab. S4 elution procedure**

| Time（min） | A ([methyl](../../../../D:/Dict/8.8.1.0/resultui/html/index.html#/javascript:;) [alcohol](../../../../D:/Dict/8.8.1.0/resultui/html/index.html#/javascript:;))（%) | B ([acetonitrile](../../../../D:/Dict/8.8.1.0/resultui/html/index.html#/javascript:;))（%) | C (0.1%Phosphoric acid wate)（%） |
| --- | --- | --- | --- |
| 0 | 0 | 6 | 94 |
| 15 | 0 | 38 | 62 |
| 30 | 0 | 38 | 62 |
| 33 | 0 | 53 | 47 |
| 63 | 0 | 55 | 45 |
| 68 | 1 | 67 | 32 |
| 78 | 1 | 85 | 14 |
| 95 | 3 | 85 | 12 |

**Tab. S5 [chemical](../../../../C:/Users/%25E9%2599%2588%25E6%2595%25AC%25E5%25BF%25A0/AppData/Local/youdao/dict/Application/8.9.3.0/resultui/html/index.html#/javascript:;) [component](../../../../C:/Users/%25E9%2599%2588%25E6%2595%25AC%25E5%25BF%25A0/AppData/Local/youdao/dict/Application/8.9.3.0/resultui/html/index.html#/javascript:;)  content of *C. migao* fruit from different fruit stages**

| **fruit stages** | **Sample** | **α_Terpineol (mg·g^-1^）** | **Sabinene (mg·g^-1^)** | **Total sugar TS(%)** | **Reducing sugar RS(%)** | **Soluble polysaccharide SP(%）** | **Crude polysaccharide CP(%)** | **Crude fat CF(%)** |
| --- | --- | --- | --- | --- | --- | --- | --- | --- |
| **young fruit**  **period(June)** | BP-1 | 10.51±0.81a | 8.34±0.96a | 11.0±1.00ab | 3.07±0.28ab | 3.15±0..15ab | 4.15±0.17a | 1.03±0.66a |
|  | LD-1 | 11.82±0.89c | 4.60±0.21d | 11.15±2.15b | 3.15±0.39ad | 3.54±0.13b | 2.05±0.02b | 2.15±0.02a |
|  | XL-1 | 14.50±1.02 | 16.08±1.00b | 7.05±1.08c | 3.17±0.39d | 4.02±0.05b | 4.15±0.11ac | 2.06±0.06a |
| **Close to maturity**  **[period](../../../../C:/Users/%25E9%2599%2588%25E6%2595%25AC%25E5%25BF%25A0/AppData/Local/youdao/dict/Application/8.9.3.0/resultui/html/index.html#/javascript:;) (August)** | BP-2 | 29.13±1.20d | 5.76±0.52d | 18.05±0.81d | 4.80±0.35ac | 2.16±0.85a | 313±0.05bc | 11.15±0.08b |
|  | LD-2 | 11.41±0.73c | 7.83±0.11e | 17.09±0.53de | 4.15±0.1c | 2.08±0.08a | 1.15±0.07e | 12.31±0.13b |
|  | XL-2 | 12.54±0.96c | 17.34±0.89b | 17.08±0.71d | 3.53±0.04d | 1.08±0.03a | 1.08±0.11B | 13.04±0.09bd |
| **[mature](../../../../C:/Users/%25E9%2599%2588%25E6%2595%25AC%25E5%25BF%25A0/AppData/Local/youdao/dict/Application/8.9.3.0/resultui/html/index.html#/javascript:;) [period](../../../../C:/Users/%25E9%2599%2588%25E6%2595%25AC%25E5%25BF%25A0/AppData/Local/youdao/dict/Application/8.9.3.0/resultui/html/index.html#/javascript:;)**  **(October)** | BP-3 | 19.42±2.11e | 5.88±0.96d | 9.02±1.02ac | 4.08±0.1abc | 10.08±0.01c | 9.15±0.7f | 30.06±5.09c |
|  | LD-3 | 8.20±0.82b | 14.04±0.56b | 15.11±1.08de | 4.32±0.1c | 12.09±0.07d | 11.03±0.54g | 36.03±0.04e |
|  | XL-3 | 7.61±0.57b | 23.22±0.21c | 14.51±0.08e | 4.15±0.01abc | 6.07±0.01e | 6.13±0.16d | 18.20±0.28d |

**Tab. S6 [chemical](../../../../C:/Users/%25E9%2599%2588%25E6%2595%25AC%25E5%25BF%25A0/AppData/Local/youdao/dict/Application/8.9.3.0/resultui/html/index.html#/javascript:;) [component](../../../../C:/Users/%25E9%2599%2588%25E6%2595%25AC%25E5%25BF%25A0/AppData/Local/youdao/dict/Application/8.9.3.0/resultui/html/index.html#/javascript:;)  content of *C. migao* fruit from different minor watershed**

| **Minor**  **watershed** | **Sample** | **α_Terpineol (mg·g^-1^）** | **Sabinene （mg·g^-1^）** | **Total sugar TS(%)** | **Reducing sugar RS(%)** | **Soluble polysaccharide SP(%）** | **Crude polysaccharide CP(%)** | **Crude fat CF(%)** |
| --- | --- | --- | --- | --- | --- | --- | --- | --- |
| **Hongshui**  **river** | LY | 19.59±0.20c | 5.31±0.02f | 25.02±0.85c | 14.04±0.12a | 1.24±0.03a | 4.05±0.03ab | 23.40±0.07c |
|  | TE | 23.55±0.38b | 5.31±0.04f | 22.09±2.28a | 14.15±0.21a | 1.60±0.05a | 2.75±0.15c | 27.53±0.16b |
|  | WM | 26.07±0.18a | 5.31±0.05f | 23.40±0.53a | 8.72±0.07B | 1.69±0.03a | 3.67±0.06b | 35.19±0.04a |
|  | CH | 13.43±0.13E | 6.75±0.07D | 22.77±0.39a | 2.83±0.04d | 2.92±0.07ab | 3.38±0.05b | 26.57±0.52b |
| **Yujiang**  **river** | FN | 13.91±0.21E | 2.96±0.04g | 24.92±0.53ac | 12.25±0.19a | 1.39±0.03a | 4.11±0.06ab | 28.03±0.39b |
|  | NP | 23.02±0.12b | 5.31±0.03f | 24.85±0.67ac | 2.68±0.06d | 1.91±0.04a | 4.02±0.01ab | 26.12±0.48b |
| **Panjiang**  **river** | ZF | 3.51±0.06f | 5.31±0.06f | 22.44±0.21a | 8.48±0.08B | 3.84±0.02b | 3.29±0.04c | 22.49±0.14c |
|  | LD | 16.44±0.22d | 6.71±0.01D | 23.28±0.45a | 5.99±0.03c | 2.25±0.02a | 5.58±0.02a | 20.89±0.39c |
|  | LB | 15.8±0.32d | 5.97±0.04e | 24.92±0.15ac | 7.90±0.02Bc | 2.64±0.03a | 3.81±0.01b | 36.97±0.41a |

**Tab. S7 Climatic characteristics of the nine collections sites**

| **Minor**  **watershed** | Sample | Maximum temperature | Minimum temperature | Annual average temperature | Average relative humidity | Annual rainfall |
| --- | --- | --- | --- | --- | --- | --- |
|  |  |  |  |  |  |  |
| **Hongshui**  **river** | Guizhou·Wangm（WM） | 35.9533 | -5.01878 | 20.2872 | 69.1774 | 1362.62 |
|  | Guizhou·Ceheng（CH） | 34.8903 | -5.45727 | 19.2473 | 70.6263 | 1318.75 |
|  | Guangxi·Tiane（TE） | 35.7399 | -4.78615 | 19.6655 | 72.3385 | 1332.93 |
|  | Guangxi·Leye（LY） | 35.6013 | -4.69967 | 19.6846 | 72.0917 | 1352.56 |
| **Yujiang**  **river** | Yunan·Funing（FN） | 33.5053 | -5.99995 | 19.7667 | 71.8118 | 1268.36 |
|  | Yunan·Napo（NP） | 33.1869 | -6.65204 | 19.8442 | 71.7932 | 1230.57 |
| **Panjiang**  **river** | Guizhou·Zhenfeng（ZF） | 33.6116 | -6.11959 | 17.9782 | 71.5254 | 1265.46 |
|  | Guizhou·Luodian（LD） | 36.2486 | -5.25723 | 19.7391 | 72.0095 | 1246.89 |
|  | Guizhou·Libo（LB） | 35.8194 | -5.91388 | 18.8502 | 73.0736 | 1462.51 |

**Tab. S8 Significance of climate factors and soil factor relative**

| **Sample** | **Maximum temperature** | **Minimum temperature** | **Annual average temperature** | **Average relative humidity** | **Annual rainfall** |
| --- | --- | --- | --- | --- | --- |
| **S-CAT** | -0.2 | 0.02 | 0.08 | -0.06 | 0.13 |
| **S-UE** | -0.47* | -0.4 | -0.03 | 0.15 | -0.21 |
| **S-ACP** | 0.1 | -0.16 | -0.51* | 0.31 | -0.08 |
| **pH** | 0.13 | -0.08 | 0.23 | -0.33 | -0.25 |
| **TK** | 0.26 | 0.22 | -0.08 | -0.3 | 0.27 |
| **AK** | -0.2 | -0.29 | -0.02 | -0.05 | 0.03 |
| **TN** | 0.25 | 0.01 | -0.05 | 0.03 | -0.12 |
| **AK** | 0.46* | 0.01 | -0.23 | 0.11 | -0.15 |
| **TP** | 0.23 | -0.06 | 0 | 0.09 | -0.17 |
| **AP** | -0.09 | -0.17 | 0.01 | -0.2 | -0.05 |

* represents a significant correlation

**Fig. S1 The OTU and Shannon index dilution curves of rhizosphere soil fungal community in *C.migao*;** Dilution curve is to randomly select a certain number of sequences from the samples, count the alpha diversity index of the corresponding samples of these sequences, draw the curve with the amount of data extracted as the abscissa and the value of alpha diversity index as the ordinate, and judge whether the data amount of this sequencing is sufficient according to whether the curve is smooth or not.

**Fig. S2 Heatmap of fungal in Rhizosphere Soil of *C.migao***; (A), (B) Phylum; (C), (D) Class; (E), (F) Order; (G), (H)Family; (I), (J)Species( exclude "unclassifired" taxa)..

**Fig. S3 Network analysis(two-factor).** The red line represents a positive correlation and the green line represents a negative correlation; (A), different small watersheds (B), different fruit periods.**Fig. S4 Kruskal-wallis rank sum test.** (a), (different small watersheds); (b), (different fruit periods); *representative the *p*＜0.05.

**Fig. S5 Network analysis(two-factor).** The red line represents a positive correlation and the green line represents a negative correlation; (different small watersheds ); (B), (different fruit period); Maximum temperature(Max-T), Minimum

temperature(Min-T), Annual average temperature(AAT), Average relative humidity(ARH), Annual rainfall(AR).

**
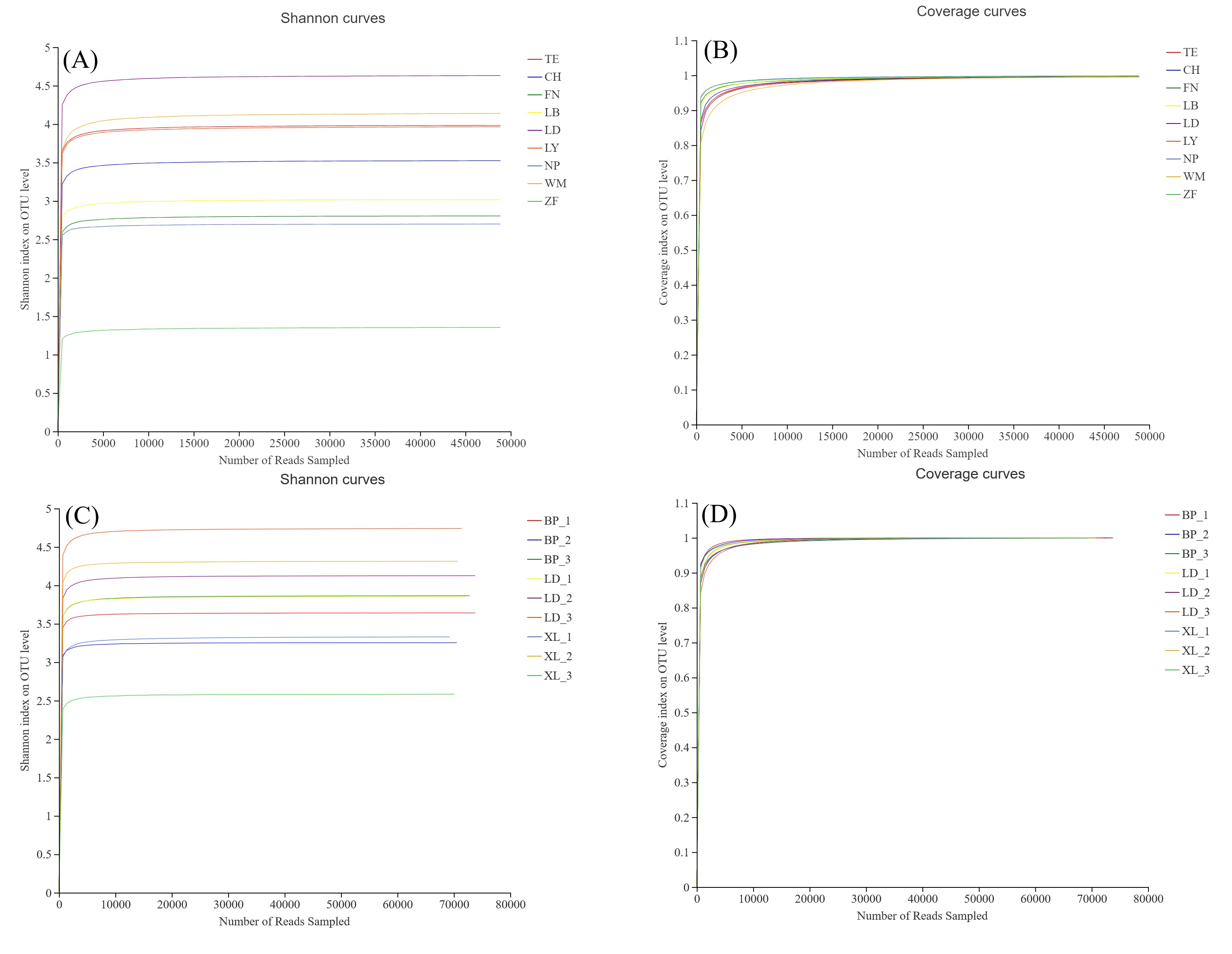
Fig. S1**


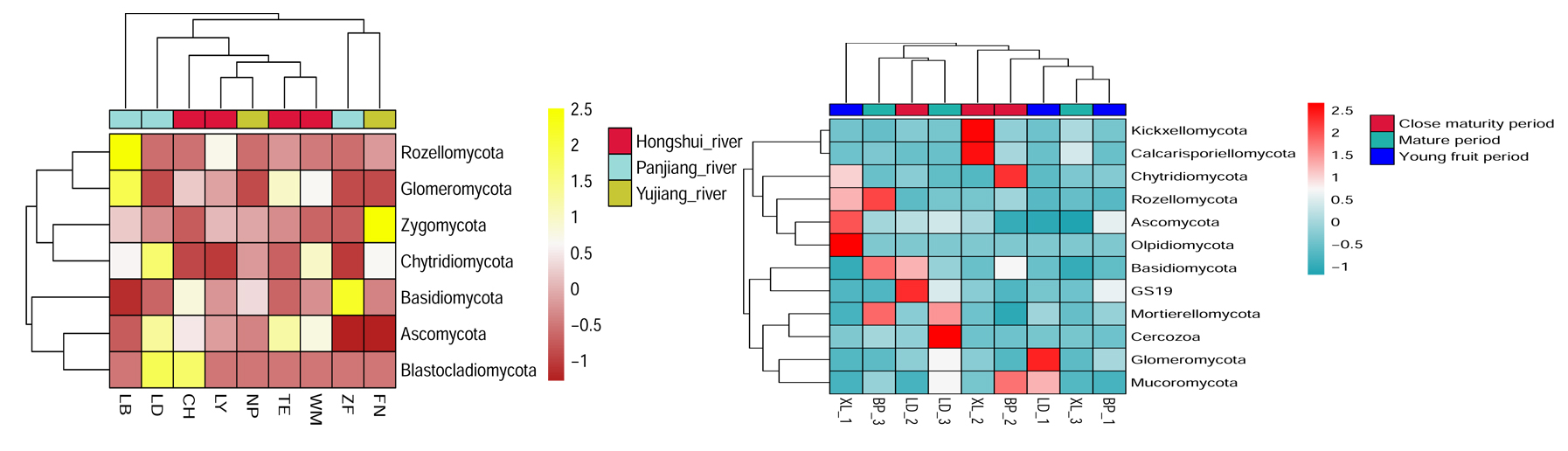

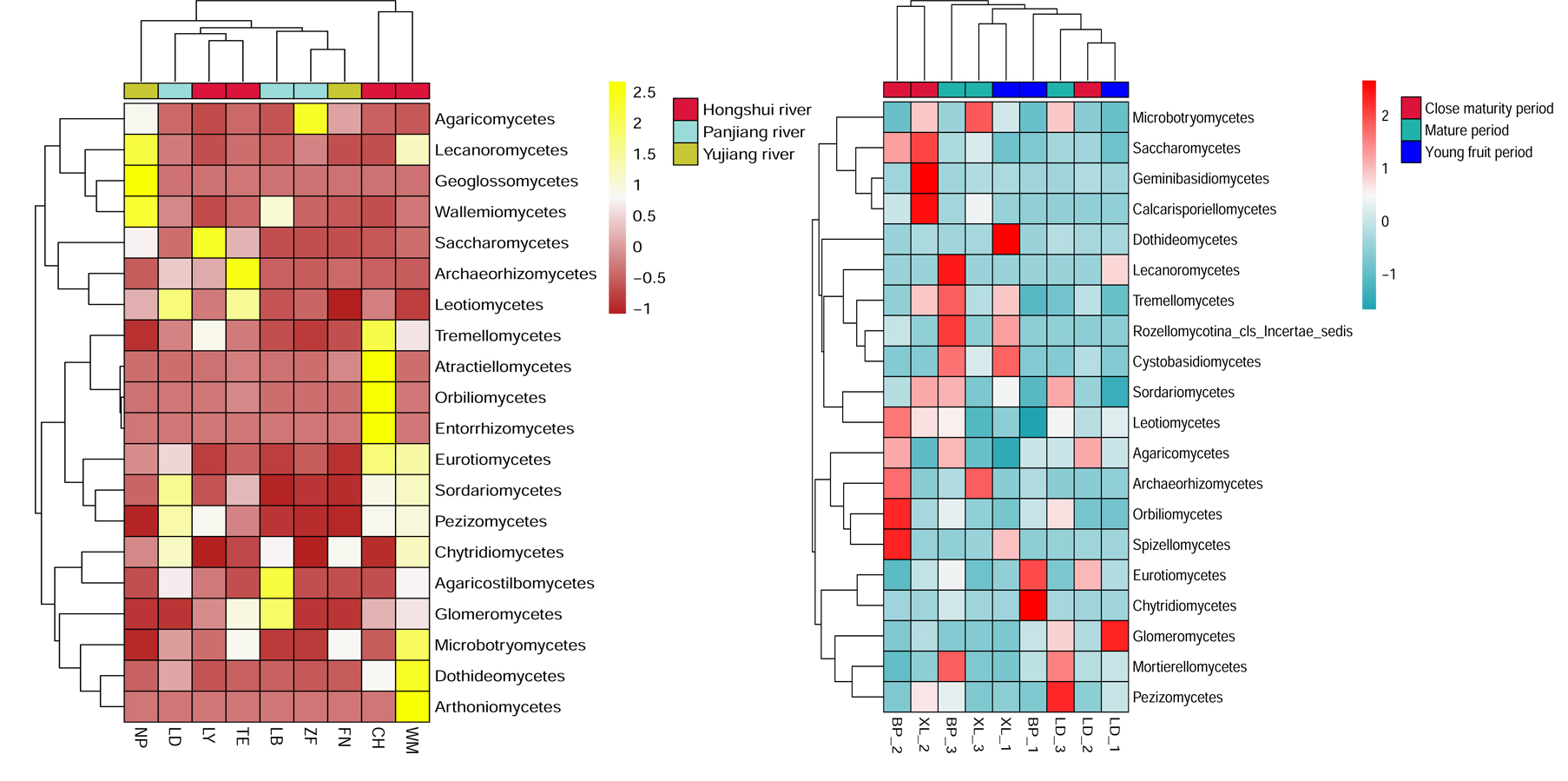

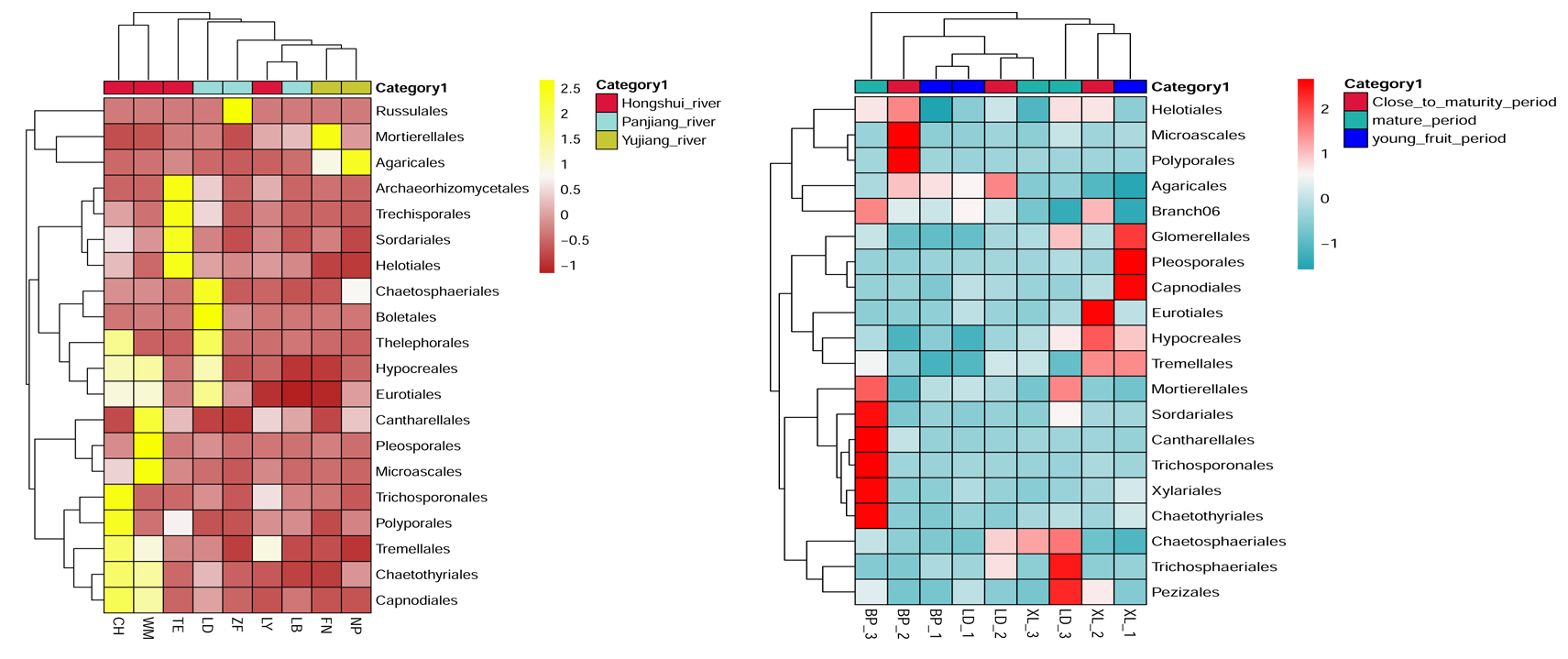

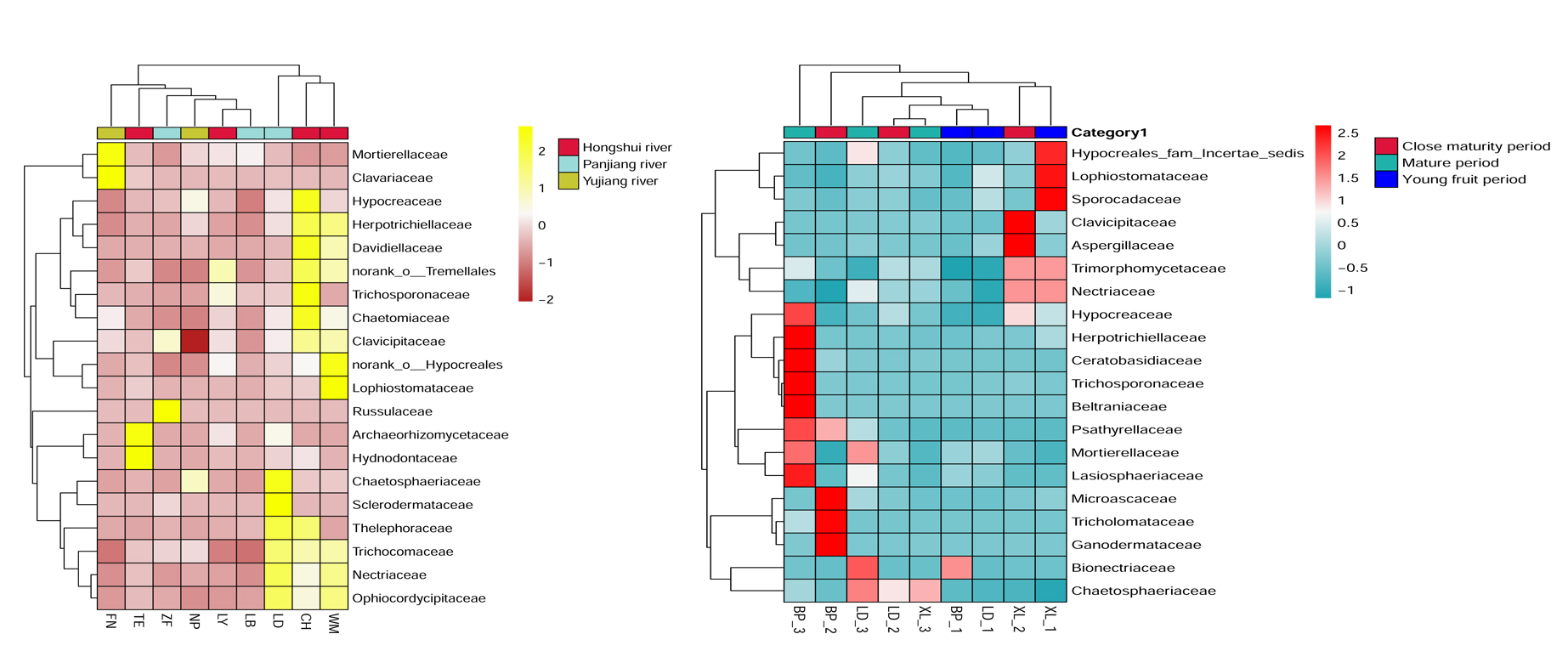

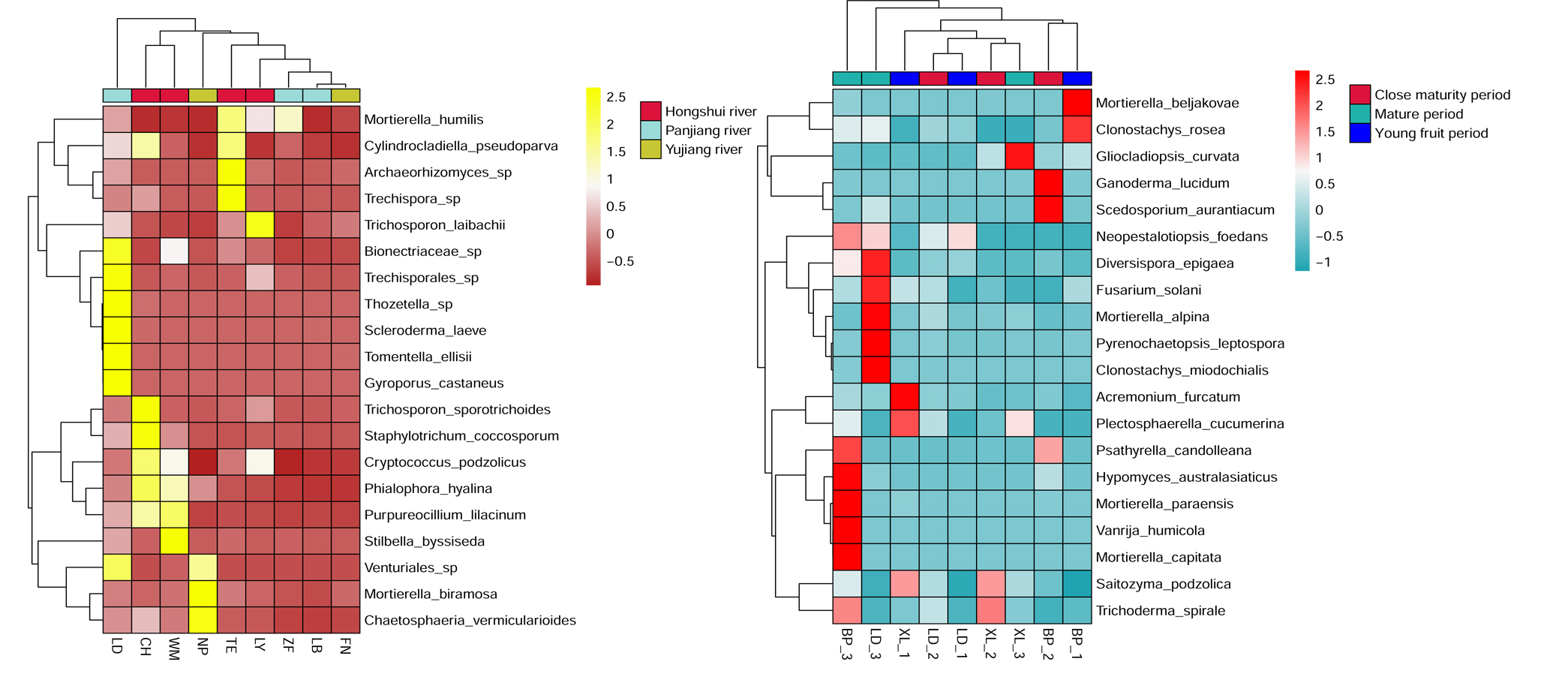


(A)

(B)

(I)

(J)

(G)

(H)

(E)

(F)

(C)

(D)

**Fig. S2**


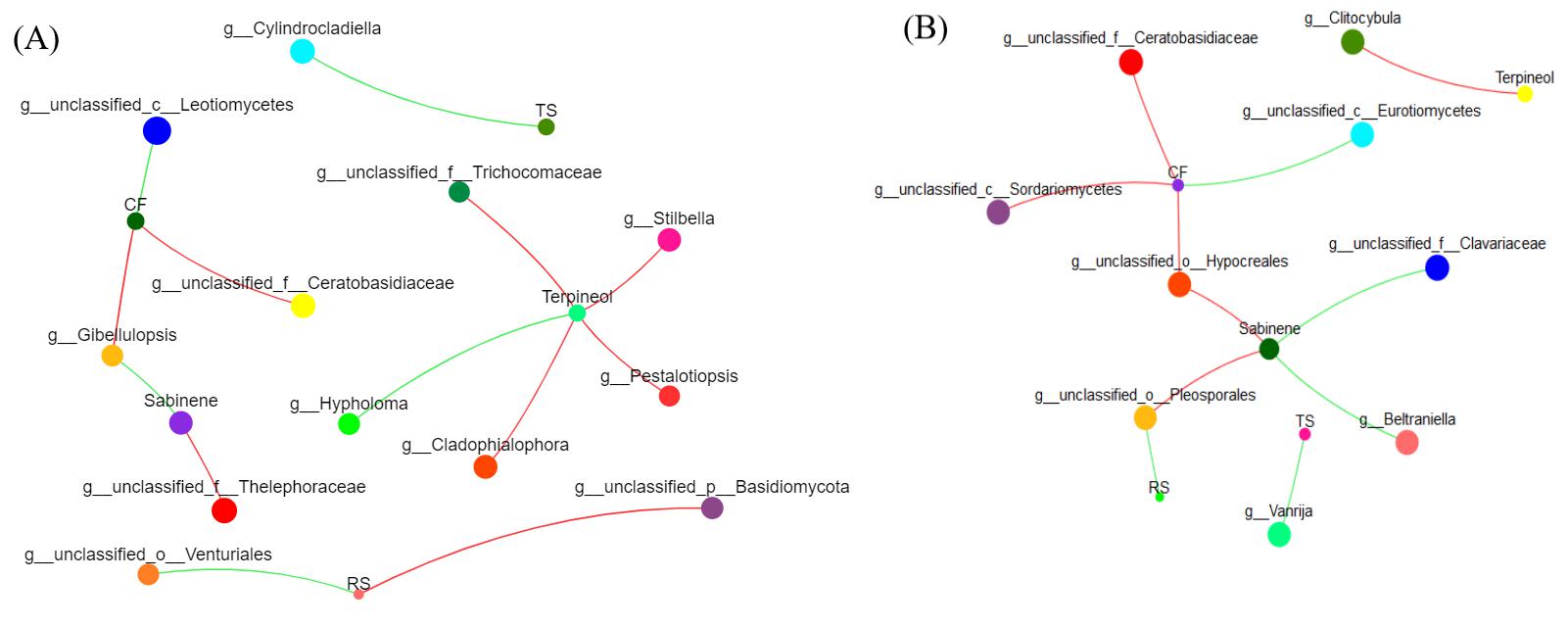
**Fig. S3**

**
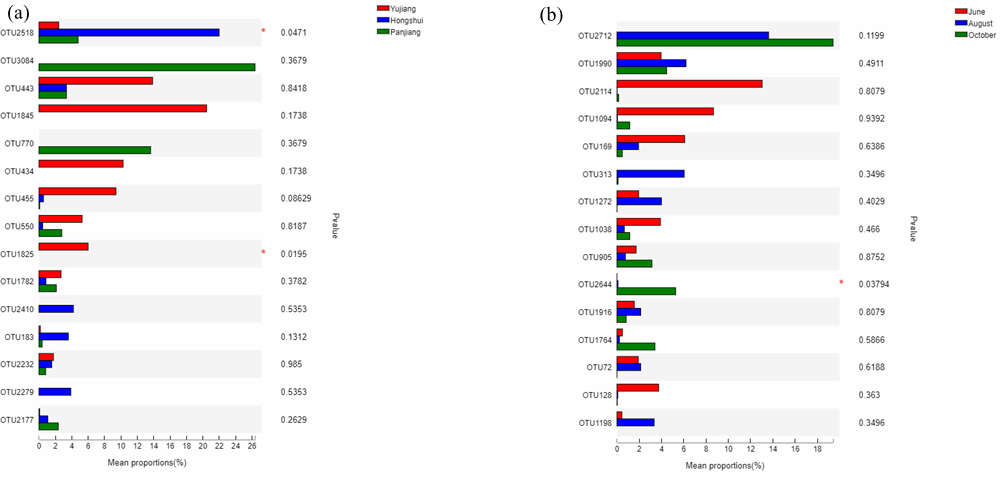
**

**Fig. S4**


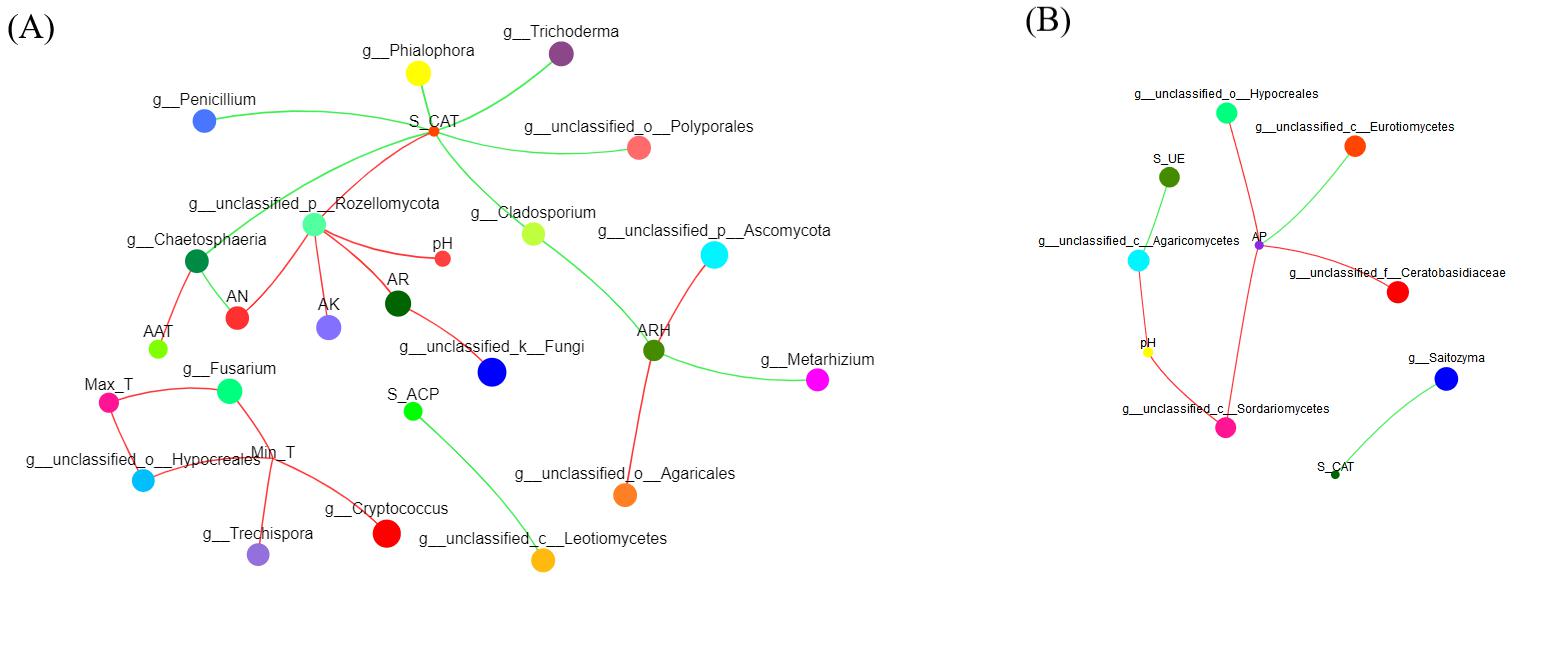
**Fig. S5**
